# FastKnock: an efficient next-generation approach to identify all knockout strategies for strain optimization

**DOI:** 10.1186/s12934-023-02277-x

**Published:** 2024-01-29

**Authors:** Leila Hassani, Mohammad R. Moosavi, Payam Setoodeh, Habil Zare

**Affiliations:** 1 Department of Computer Science and Engineering and IT, School of Electrical and Computer Engineering, Shiraz University, Shiraz, Iran; 2 Department of Chemical Engineering, School of Chemical, Petroleum and Gas Engineering, Shiraz University, Shiraz, Iran; 3 Booth School of Engineering Practice and Technology, McMaster University, Hamilton, ON Canada; 4 Department of Cell Systems and Anatomy, University of Texas Health Science Center, San Antonio, TX USA; 5 Glenn Biggs Institute for Alzheimer’s & Neurodegenerative Diseases, University of Texas Health Science Center, San Antonio, USA

**Keywords:** Genome-scale metabolic model, Reaction knockout strategy, Growth-coupled biosynthesis, Biochemical overproduction, Mathematical optimization, Reaction clustering, Search space reduction

## Abstract

Overproduction of desired native or nonnative biochemical(s) in (micro)organisms can be achieved through metabolic engineering. Appropriate rewiring of cell metabolism is performed by making rational changes such as insertion, up-/down-regulation and knockout of genes and consequently metabolic reactions. Finding appropriate targets (including proper sets of reactions to be knocked out) for metabolic engineering to design optimal production strains has been the goal of a number of computational algorithms. We developed FastKnock, an efficient next-generation algorithm for identifying all possible knockout strategies (with a predefined maximum number of reaction deletions) for the growth-coupled overproduction of biochemical(s) of interest. We achieve this by developing a special depth-first traversal algorithm that allows us to prune the search space significantly. This leads to a drastic reduction in execution time. We evaluate the performance of the FastKnock algorithm using various *Escherichia coli* genome-scale metabolic models in different conditions (minimal and rich mediums) for the overproduction of a number of desired metabolites. FastKnock efficiently prunes the search space to less than 0.2% for quadruple- and 0.02% for quintuple-reaction knockouts. Compared to the classic approaches such as OptKnock and the state-of-the-art techniques such as MCSEnumerator methods, FastKnock found many more beneficial and important practical solutions. The availability of all the solutions provides the opportunity to further characterize, rank and select the most appropriate intervention strategy based on any desired evaluation index. Our implementation of the FastKnock method in Python is publicly available at https://github.com/leilahsn/FastKnock.

**Supplementary Information:**

The online version contains supplementary material available at 10.1186/s12934-023-02277-x.

## Introduction

Metabolic engineering aims at the proper rewiring of cell metabolism to construct genetically engineered strains that can serve as robust cell factories for a variety of purposes, including the biosynthesis of target substances [1]. Extensive studies have been conducted in this field to develop methods for efficiently producing suitable natural compounds by using either native cells or heterologous hosts [2, 3]. Systems metabolic engineering employs the concepts and capabilities of systems biology, synthetic biology, and evolutionary engineering at the systems level. It uses approaches from these disciplines and combines them with standard metabolic engineering techniques to facilitate the development of high-performance strains [4–7]. Metabolic systems biology plays a significant role in systems metabolic engineering because it incorporates a systems-level perspective on cellular metabolic functionalities [8–11]. Using metabolic systems biology, scholars can integrate omics data with results from genome-scale computational simulations to improve metabolic engineering techniques. These techniques can lead to the development of potentially productive and operationally optimized microbial strains [10–13].

The growth-coupled overproduction of (bio)chemicals is one of the most vital and practical objectives in systems metabolic engineering. Using this approach, synthesis of a desired compound can be guaranteed along with the reproduction of the engineered cell(s) [14, 15]. Genome-scale metabolic network reconstructions (GENREs) [16] and their relevant mathematical representatives (genome-scale metabolic models (GEMs)) have been developed for numerous microorganisms (e.g., *Escherichia coli* [17–20], *Pseudomonas putida* [21, 22], and *Saccharomyces cerevisiae* [23–26]). These tools are commonly used in computational systems biology for in silico production strain design. In particular, biased COnstraint-Based Reconstruction and Analysis (COBRA) computational techniques such as flux balance analysis (FBA) [27] and flux variability analysis (FVA) [28] are useful in analyzing GEMs [11, 12, 29, 30] (Additional file 1: Supplement A). Using COBRA, one can take advantage of the synergistic effects of a variety of basic elements including genes, gene products and metabolites to evaluate cells’ potential and make model-driven discoveries. Accordingly, in silico studies based on systems-level analyses inspire researchers to examine intervention strategies, including gene or reaction insertions, knockouts, and up- or down-regulations [31, 32]. For example, in several studies on gene and reaction knockouts, the candidates for the best combination of eliminations were identified [15, 33–36].

There are two basic conventional approaches for designing metabolic intervention strategies: top-down (e.g., OptKnock [33], OptGene [37], MoMAKnock [34], CiED [38]) and bottom-up (e.g., FSEOF [39], CosMos [40]) procedures [41, 42]. The top-down strategies are used to determine whether the potential interventions are advantageous and they iteratively search for the metabolic reaction network of interest until the optimal solutions are identified. The search space in the corresponding problems includes all combinations of a predefined number of reactions in a GEM. Due to the size of the developed and highly curated GEMs, this search space is extremely large and would explode with the cardinality of the combination. Thus, it would not be feasible to conduct an exhaustive exploration within a reasonable time frame.

Optimization techniques are commonly proposed to address this computational challenge. For example, OptKnock [33] is one of the most popular top-down frameworks. It uses bi-level optimization for in silico metabolic engineering. It aims to identify the appropriate sets of genes or reactions that, when knocked out, maximize the production rate of the desired biochemical coupled with biomass formation. To find an optimal solution for the growth-coupled production of the biochemical(s) of interest, OptReg [31] expands the capabilities of OptKnock by predicting appropriate up- or down-regulation of revealed crucial genes or reactions. RobustKnock [43] has been developed based on optimization techniques that guarantee the minimum production rate of the desired biochemical. Despite its novel approach, RobustKnock has not been widely used due to the difficulty of implementation.

The challenge in employing these optimization approaches is that the time required for finding an optimal solution grows exponentially with the cardinality of the combination. Worse, the solvers may fall into a deadlock situation and become trapped in an infinite loop. Several metaheuristic algorithms have been proposed to overcome this obstacle. These algorithms can pinpoint the suboptimal solutions within a reasonable time. For example, BAFBA [44] is a top-down metaheuristic method that deploys the bees algorithm [45] to find candidate gene knockouts and evaluate the results through FBA (Additional file 1: Supplement A).

Bottom-up approaches discover appropriate intervention strategies by comparing two flux distributions. One of these distributions relates to the wild-type, which aims to maximize the cell’s growth rate. The other distribution relates to the functional state, which takes into account the goal of the desired biochemical overproduction. Examples include the flux distribution comparison analysis (FDCA) algorithm [46] and OptForce [32]. Using OptForce, all coordinated reaction modifications contributing to target overproduction are identified based on significant differences between the two flux patterns (initial and desired) in the introduced network, calculated using FVA. FVA finds the boundaries of the reaction fluxes that can satisfy the optimality of the solution under steady-state flux analysis (Additional file 1: Supplement A).

In a nutshell, primitive top-down approaches use optimization methods to find an optimal solution at the cost of significant execution time. While top-down metaheuristic approaches require less computational resources, they are not guaranteed to find a globally optimal solution because the search space contains many local optima. On the other hand, bottom-up approaches can be used to find a set of potential solution candidates [14]. Despite various integrated computational and experimental studies, it is challenging to identify the most proper and operative alterations by only comparing the flux distributions of the wild-type to the ideally engineered states. Considering high order cardinalities and interventions [47] adds to the complexity of the problem.

State-of-the-art approaches have been developed to dramatically alleviate the computational challenges and significantly reduce the computational costs including (iteratively) pruning the search space [48, 49] and sequentially enumerating the smallest minimal cut sets (MCSs) in order to provide several solutions [50]. For example, Fast-SL properly explores a metabolic network of interest to find the most appropriate synthetic lethal reaction sets. Fast-SL improves the performance of a brute-force search algorithm by iteratively reducing the size of the search space, which substantially shortens the execution time [49]. MCSEnumerator is another novel method that attempts to find many solutions using MCSs aimed at the identification of either synthetic lethal sets or optimal strain design targets [50].

Calculating the MCSs in GEMs is a complex and challenging computational problem [51]. The scalability of MCSEnumerator algorithms paves the way for both theoretical and practical studies considering high-order simultaneous reaction interventions for strong growth-coupled product formation [52, 53]. However, for in silico strain design, the MCSEnumerator approach require predefining of the acceptable thresholds for growth and target product yields and this contributes to different drawbacks such as neglection of some appropriate suboptimal solutions [54].

In this paper, we present FastKnock as a next-generation knockout strategy algorithm that provides the user with all possible solutions for multiple gene and reaction knockouts to overproduce a (bio)chemical of interest. Unlike the MCSEnumerator approach, FastKnock does not rely on any special parameter settings and additional assumptions (except for predefining the maximum number of simultaneous reaction knockouts). We developed a delicate search and prune algorithm to accomplish this goal at a greatly reduced computational time and cost. Our method combines (and benefits from) both basic approaches to tackle the problems described above. It incorporates reaction knockouts to couple the biosynthesis of both primary (e.g., succinate, lactate, ethanol, etc.) and secondary metabolites (e.g., dodecanoic acid, polyketides such as erythromycin and terpenoids such as lycopene) with cell reproduction. It examines the GEM at the level of metabolic reactions while checking the corresponding genes to consider the gene dependency of the reactions.

The availability of all solutions allows us to systematically characterize and rank these strategies in accordance with some criteria including (a) substrate-specific productivity (SSP) [14, 15, 55, 56], (b) strength of growth coupling (SoGC), defined as the square of the product yield per unit substrate divided by the slope of the lower edge of the production curve [14, 15, 55, 56], (c) strain dynamic performance, which depends on yield, productivity, and titer [57, 58], and (d) other important indices reflecting environmental and operational considerations such as minimal production of undesired or toxic byproducts and the feasibility of CO_2_ biofixation. Some alternative criteria are discussed in [59]. Furthermore, it would be possible to evaluate the solutions and categorize them in the different major classes: potentially, weakly, directionally growth-coupled production (pGCP, wGCP, dGCP) and substrate-uptake coupled production (SUCP) raised in [60].

The article is structured as follows: Initially, the FastKnock algorithm is introduced. Subsequently, we present the outcomes of in silico experiments utilizing meticulously curated GEMs of *E. coli*. Finally, discussions and conclusions are articulated.

## The proposed method

We developed the FastKnock algorithm, a versatile framework intended to enhance the production rate of a targeted metabolite within a cell while promoting growth. This desired metabolite may belong to either a primary or secondary category and can be of native or heterologous origin. Specifically, the algorithm can be applied to heterologous metabolites through the inclusion of the associated pathways into the GEM set.

In other words, FastKnock identifies reactions to be deleted from the network while ensuring that the flux of the biomass formation reaction remains above a specific cut-off (i.e., 1% of *gr*_*WT*_, (Additional file 1: Supplement D) and maximizes the production of the desired substance(s) [61]. For practical applications, FastKnock can be utilized to identify subsets of network reactions that can be removed to significantly enhance the production of the desired biochemical. Specifically, FastKnock identifies the strains in which the production rate of the desired biochemical surpasses a predefined threshold in the base model (i.e., the model without any interventions). We refer to this threshold as *Th*_*chemical*_, defined as 5% of the maximum theoretical yield (i.e., the optimal production rate of the desired biochemical when it is considered the objective of the cell metabolism) in the base model. FastKnock, like other common approaches, employs preprocessing to reduce the size of metabolic model reactions and the search space. In the preprocessing phase (Additional file 1: Supplement C), a subset of reactions is identified and structurally excluded from the metabolic network to generate a reduced model denoted as *Reduced_model*. Additionally, the set of candidate reactions for deletion from the model is determined and denoted as *Removable*.

The search space of the exhaustive search includes all members of the power set of the *Removable* set with a particular maximum cardinality.

The search space grows exponentially as the size of the set increases. Therefore, conducting an exhaustive search and examining all subsets of reactions is highly time-consuming and infeasible. To address this challenge, our proposed algorithm utilizes information available only during the search procedure to dynamically narrow the search space—iteratively pruning the space and temporarily excluding certain reactions. This reduced search space is employed to identify knockout strategies, and we refer to it as the *target space*.

### The FastKnock algorithm

Our proposed method aims to identify all solutions to a strain optimization problem (with a predefined maximum number of reaction deletions), enabling the growth-coupled overproduction of a metabolite (biochemical) of interest. Each solution represents a set of *k* reactions (i.e., a knockout strategy) in which the elimination of these reactions results in a new engineered strain, coupling the overproduction of the biochemical of interest with cell growth.

Testing whether a set of reactions is a proper solution is equivalent to solving an optimization problem in which the objective function is the growth of the cell and the elimination of reactions corresponds to modifying the associated constraints of the optimization problem (Additional file 1: Supplement F). By solving this optimization problem, we obtain the flux of all the reactions including the production rate of a desired biochemical. An appropriate solution (i.e., a knockout strategy) should satisfy the objective function along with providing a suitable production rate for the desired biochemical product.

**Fig. 1:**
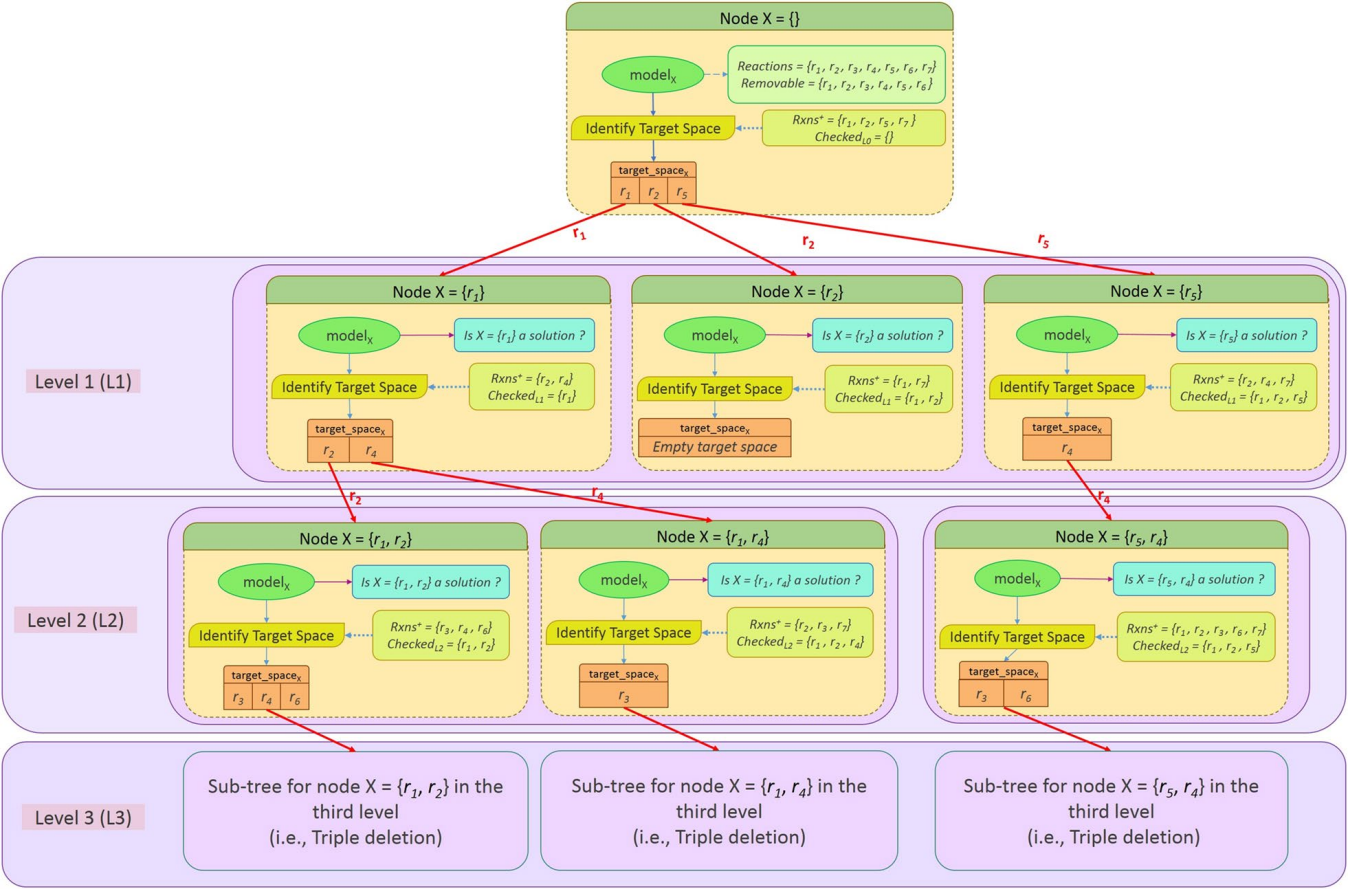
The traversal tree: All possible solutions are identified through a depth-first traversal of the tree. First, the *identifyTargetSpace* function is applied in the root node to the reduced wild-type network to determine the target space. Each reaction in this set is individually selected and removed from the network in Level 1. For each deleted reaction (or equally node) in Level 1, the *identifyTargetSpace* function is recalled to obtain the target space for the next level. For simplicity, we show only two levels of the traversal of the tree, which is enough to identify all single and double deletions

To find all reaction subsets of size ≤ *k*, we employ a tree-based representation that encompasses all combinations of reactions with a maximum size of *k*, as outlined below. Figure 1 illustrates the overall procedure using a depth-first traversal tree. The root node at level zero corresponds to the base model in which no reaction is deleted (i.e., the reduced model). All sets of *k* reactions are placed in nodes of the tree in depth *k* (i.e., at the level *k*). The FastKnock procedure starts with investigating the elimination of a single arbitrary reaction *r*_*1*_ at level one. Whether knocking out *r*_*1*_ is a solution or not, we proceed to explore the simultaneous elimination of *r*_*1*_ and another reaction at level two. At each level, we consider only the reactions with nonzero flux, determined by the optimization problem solved in the parent node at the upper level (Additional file 1: Supplement F, part 2). The procedure of adding reactions with nonzero flux to the set of knockout reactions continues at lower levels of the tree until one of the two stopping conditions is met: a) we reach a leaf at level *k* (the predefined number of knockouts), or b) we reach a node guaranteed to have no solution in its subtree.

To check condition b in each node at level *l < k*, we determine whether the subtree may lack a solution by investigating the optimization problem. If the optimization problem already indicates an infeasible region at a node, adding more constraints in the subtree of the node would not lead to a proper solution (see Additional file 1: Supplement F).

The merit of the procedure is the technique of bounding the search by a) excluding the reactions with zero flux at each node from the target space of the node (Additional file 1: Supplement F, part 2) and b) checking the feasibility of reaching a solution *before* expanding the subtree of each node. If a reaction has zero flux based on the functional state of a node in the traversal tree, it is excluded from the target space of that node. However, in the children of that node, the functional states may change and the reaction can get nonzero flux. Thus, it might reappear in the search space when we explore the descendants at consequent levels. This dynamic and effective pruning of the search space enhances the efficiency of the algorithm.

Algorithm 1 represents the definition of a node in the tree, as well as, the main procedure of the FastKnock algorithm. Each instance of the *Node* contains the model, the set of the removed reactions, the search space, and the target space for the next level (Fig. 1). Specifically, at each node *X* of the tree at level *L*, we investigate a set of *L* reactions (*deleted_rxns*) to determine (a) whether *X* is a solution and (b) the new target space, which is the set of all reactions that could potentially be added to *deleted_rxns* for investigation at the next level.

Determining the target space at each node is critical, and it allows us to avoid the combinatorial explosion of the tree that would inevitably result from an exhaustive search. In particular, while we investigate drastically fewer subsets of reactions at the children nodes in Level *L* + 1, our analysis guarantees that FastKnock will find every candidate solution (Additional file 1: Supplement F).

In Algorithm 1, the traversal of the tree shown in Fig. 1 is represented by a set of queues: *queue*_*1*_ to *queue*_*target_level*_. Each queue contains a set of nodes. At each moment during the execution of the algorithm, queue *l* contains all children of a certain node at level *l*-1 being investigated. In this way, the subtrees are gradually constructed and removed (pruned).

#### Algorithm 1:

The FastKnock main procedure


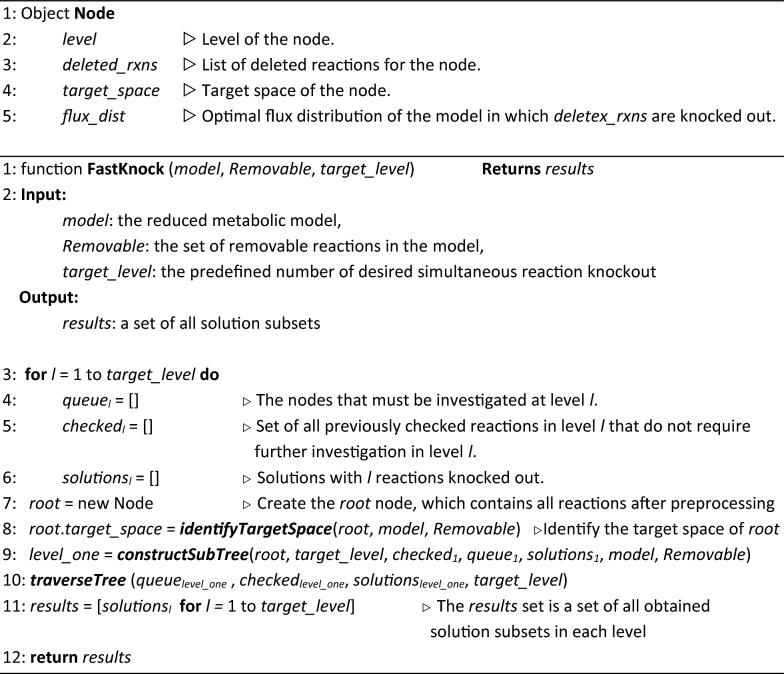


The main algorithm consists of three functions: *identifyTargetSpace*, *constructSubTree*, and *traverseTree*. For each node, we compute a target space and a flux distribution using the *identifyTargetSpace* function. This function temporarily narrows the search space for the whole subtree of the node. The subtree of a node is constructed using the *constructSubTree* function. The *traverseTree* function recursively navigates the tree, based on a depth-first traversal.

We elaborate on these functions in the following subsections. Firstly, we determine the target space and subsequently describe the search procedure—detailing how the traversal tree is partially constructed and traversed. In our implementation, we enhanced the quality of the obtained solutions by ensuring a minimal chemical production rate (Additional file 1: Supplement I) and increased the speed of the algorithm through parallel processing (Additional file 1: Supplement G).

#### Identifying the target space

At steady state, a specific flux range for each reaction *r* is obtained (*minFlux*_*r*_ ≤ *f*_*r*_ ≤ *maxFlux*_*r*_), which leads to the optimal cellular objective (e.g., maximizing the biomass formation flux). Knocking out a reaction *r* is implemented by setting the allowable flux range [62] of the reaction to zero (i.e., lb_r_ = ub_r_ = 0 in the optimization problem of Equations a.1 and a.5 in Additional file 1: Supplement A). Note that when a reaction is reversible (i.e., the obtained flux range of a reaction includes zero minFlux_r_ ≤ 0 ≤ maxFlux_r_), knocking out that reaction alone has no effect on the optimal linear objective value of the network in FBA (Additional file 1: Supplement F).

Here, the main idea is to prune the target space by considering only the set of reactions with nonzero flux values. This approach significantly reduces the size of the target space and thus reduces the execution time of the algorithm.

We denote reactions that lack a zero value in their obtained flux range as *Rxns*^+^ in each node of the tree:

##### ${Rxns}^{+}=\{r \in Rxns ~|~{minFlux}_{r}>0 ~~~or~~~ {maxFlux}_{r}<0\}.$

The target space of each node, which is the set of reactions that could be appropriate for deletion, is obtained using the *identifyTargetSpace* function (Algorithm 2). The search operation at each node is limited to *Rxns*^+^  ∩ *Removable*, as shown in Line 6 of Algorithm 2.

It is worth mentioning that by any manipulation of the model, the fluxes of other reactions may change. Therefore, the functional states (i.e., flux distributions) should be analyzed repeatedly after each modification (i.e., after each reaction knockout) using FBA to identify the reactions that carry nonzero flux in the network (*model*_*X*_) (Lines 4–5). The *flux_dist* variable of the node is updated at Line 4. The intersection of these reactions and the *Removable* set construct the target space of node *X* in Line 6.

###### Algorithm 2:

Identifying target space for each node


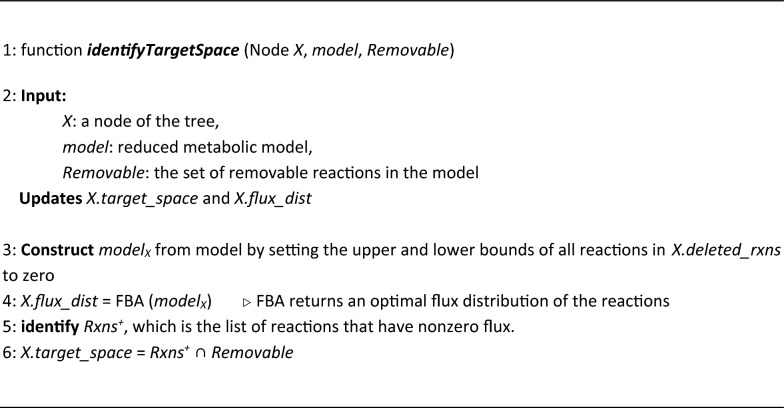


#### The search procedure

Here, we introduce a depth-first search procedure based on the traversal tree shown in Fig. 1. Each node of the tree has its own subtree, which is traversed before moving on to its sibling nodes. This *depth-first search procedure* is implemented using the *traverseTree* function of Algorithm 3.

In each call, the *traverseTree* function visits a certain node *X* (i.e., the first node of the *queue*_*level*_) and, if needed, calls the *constructSubTree* function to create the corresponding subtree of the node (Algorithm 4). The *constructSubTree* function creates the children nodes of *X*, which is a set of nodes that are placed in *level* = X.*level* + 1. For each child, *deleted_rxn*s is initialized by adding one of the reactions in *X.target_space* to the *X.deleted_rxns*.

It is clear that the order of the knocked-out reactions is not important. In FastKnock, repetitive permutations of the reactions are ignored using a *checked*_*level*_ queue for each level of the tree. Generally, *N* levels are considered for simultaneously knocking out *N* reactions from the cell. Precisely, the reaction selected for the *i*^*th*^ level is not allowed in the *(i* + *1)*^*th*^ to *N*^*th*^ levels. To generate all combinations of these reactions, the *checked*_*L*_ queue is used at level *L*. At level *L*, by deleting a reaction *r* from the target space, *r* is added to the *checked*_*L*_. This excludes the reaction from the target space of the subsequent levels.

##### Algorithm 3:

Traversing the tree


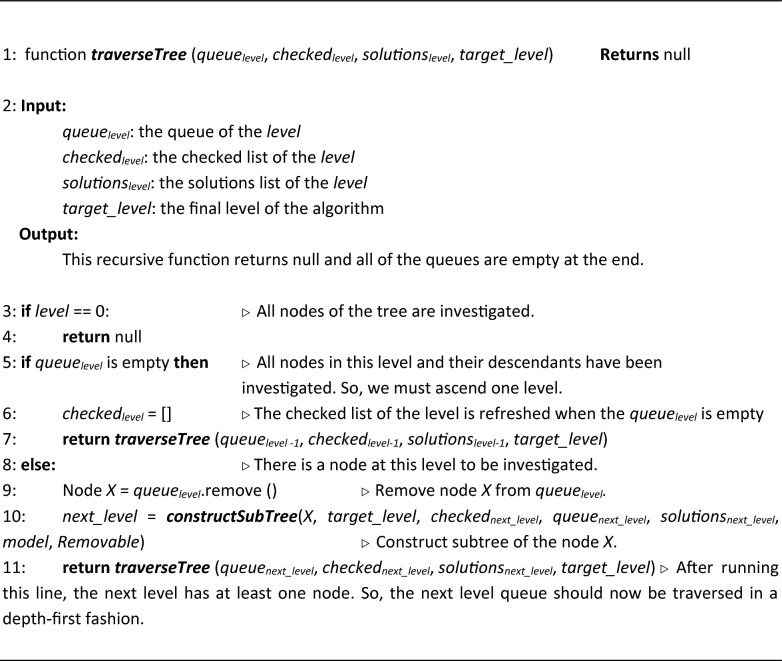


##### Algorithm 4:

Constructing subtrees of the traversal tree


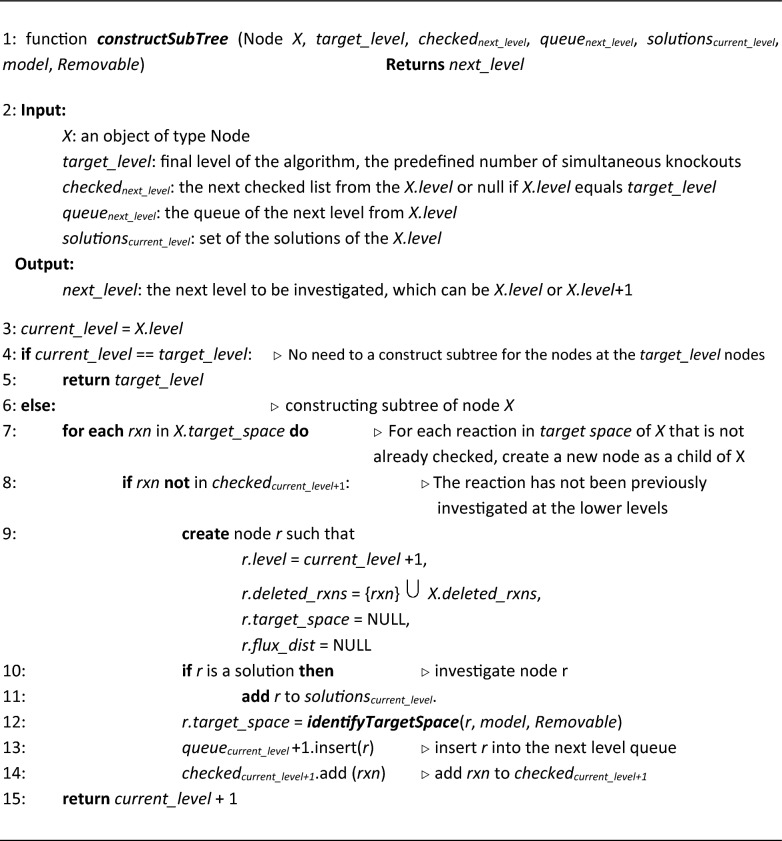


#### A traversal example

To illustrate the formation of the traversal tree, a sample node of Fig. 1 is explained here. Consider node *X* = {*r*_*1*_, *r*_*4*_} representing a double knockout of the reactions *r*_*1*_ and *r*_*4*_. Deletion of the reaction *r*_*1*_ as a single reaction knockout strategy has been checked in the parent node {*r*_*1*_} beforehand. Also, double knockout of the reactions *r*_*1*_ and *r*_*2*_ and triple knockout of {*r*_*1*_, *r*_*2*_*, r*_*3*_}, {*r*_*1*_*, r*_*2*_*, r*_*4*_}, and {*r*_*1*_*, r*_*2*_*, r*_*6*_} have been checked in the sibling node {*r*_*1*_*, r*_*2*_} and its children nodes before visiting node *X*. Visiting node *X* corresponds to checking the removal of {*r*_*1*_*, r*_*4*_} as a potential knockout strategy. Afterward, its subtree is generated to investigate the simultaneous removal of all the subsets of the removable reactions along with *r*_*1*_ and *r*_*4*_.

Naively, for each reaction in the removable set, we should generate a child node for *X* (obviously except for the reactions *r*_*1*_ and *r*_*4*_). As mentioned in the root node of Fig. 1 in this example, the set of removable reactions is supposed to be {*r*_*1*_*, r*_*2*_*, r*_*3*_*, r*_*4*_*, r*_*5*_*, r*_*6*_}. In a very simple search procedure, node *X* would have four child nodes (i.e., {*r*_*1*_*, r*_*4*_*, r*_*2*_}, {*r*_*1*_*, r*_*4*_*, r*_*3*_}, {*r*_*1*_*, r*_*4*_*, r*_*5*_}, {*r*_*1*_*, r*_*4*_*, r*_*6*_}). Generally in an exhaustive search, for each node, we may have too many children nodes and such a branching factor leads to a large search space and hence an excessive runtime.

In FastKnock, the size of the target space determines the number of children nodes of *X*, which is limited to *Rxns*^+^  ∩ *Removable*, where *Rxns*^+^ consists of nonzero flux reactions (suppose {*r*_*2*_*, r*_*3*_*, r*_*7*_} for node *X*). Because the reaction *r*_*2*_ is checked in the subtree of the sibling node {*r*_*1*_,* r*_*2*_} (see *checked*_*L2*_ = {*r*_*1*_*, r*_*2*_*, r*_*4*_} in node *X*), and the reaction *r*_*7*_ does not exist in the removable set of the model, the target space of node *X* contains only *r*_*3*_. In this way, the search space is drastically narrowed down by generating a limited number of children.

In this example, the reaction *r*_*5*_ does not exist in the *Rxns*^+^ of node *X*, due to its zero flux. It means that the node {*r*_*1*_*, r*_*4*_*, r*_*5*_} will not be added as a child of *X*, because it produces the same conditions as exist in node *X* (i.e., the same target space that results in a duplicate node). As discussed in Part 2 of Supplement F, no feasible solution would be missed because of this search space reduction (See Additional file 1: Supplement F).

It should be noted that the target space is temporarily reduced and its size may increase in the descendant nodes. In the node {*r*_*1*_*, r*_*4*_*, r*_*3*_}, the set of nonzero flux reactions could include any of the reactions in the model.

### Co-knockout of the reactions

For practical applications, one important feature of FastKnock is that it can optionally consider genes as the basis of candidate reactions for deletion. This is a realistic assumption because knocking out genes to remove a specific reaction often leads to removing a predetermined set of reactions that are simultaneously knocked out.

In fact, a reaction cannot be removed from a living cell while its genes are being manipulated in vivo. Therefore, the mapping of reactions to genes should be considered in the algorithm to reach realizable results. In other words, a reaction is knocked out from the network based on its associated gene rule. Furthermore, the clustering of reactions based on the associated gene rules could improve the efficiency of the search procedure for finding the appropriate targets.

In the simplest form of gene rules, a reaction could be removed by knocking out at least one gene from a set of genes (logical AND relation) or by simultaneously deleting a set of genes (logical OR relation). However, in general form, gene rules describe complex relationships between genes and reactions. Thus, well-known knockout strategies for in silico strain design are based on reactions or genes but do not simultaneously consider both of them.

For capturing the complexity of gene-reaction relationships, in this work, we label a set of reactions as *co-knocked out* if they are removed due to the elimination of a single gene. In the preprocessing phase of the proposed framework, for each reaction *r*, a set of reactions named *Co_KnockedOut*_*r*_ is defined that contains all the reactions that are intrinsically removed by the deletion of a set of genes that should be knocked out for removing the reaction *r*. Supplement E elaborates a modified version of the proposed algorithm based on knocking out genes rather than reactions, which discusses different forms of gene rules ﻿(See Additional file 1: Supplement E).

Although the presented method enhances time efficiency, it can be excluded from the main method to obtain comparable results with the state-of-the-art reaction-based approaches. On the other hand, this technique can be incorporated as a preprocessing step in other metabolic engineering algorithms and in silico strain design approaches.

## Results

We implemented the FastKnock algorithm using Python language programming (Version 2.7) and the COBRApy library (Version 0.15.4) [63]. We evaluated the performance of FastKnock using various examples, and we compared these results to OptKnock and MCSEnumerator approaches.

To assess FastKnock’s performance and demonstrate its capabilities while addressing potential limitations of other methods, such as the impact of model size and culture medium richness on method performance, we selected four highly-curated GEMs for *E. coli* (i.e., iJR904 [17], iAF1260 [18], iJO1366 [19], and iML1515 [20]) for our experiments. We investigated the excessive production of renowned metabolites (succinate, lactate, 2-oxoglutarate, and lycopene, functioning as both primary and secondary biological products) across various media types, including mineral and rich mediums, as diverse case studies.

We assessed the overproduction of the primary metabolites using these GEMs as wild-type models (referred to as *Strain0* in the in-silico experiments), focusing on two mineral and one rich cultivation conditions. The first condition, *CM1*, involved *i*M9 mineral medium supplemented with glucose (a maximum allowable glucose uptake rate of 10 mmol.gDW^−1^ h^−1^) under aerobic conditions (a maximum allowable oxygen uptake rate of 15 mmol.gDW^−1^ h^−1^). The second condition, *CM2*, included *i*M9 mineral medium with the same glucose supplementation (a maximum allowable glucose uptake rate of 10 mmol.gDW^−1^ h^−1^) under anaerobic conditions (an oxygen uptake rate of 0 mmol.gDW^−1^.h^−1^). In a complex and rich environment, more inputs activate cellular functions, leading to the involvement of more pathways and reactions in the network. In order to further evaluate the exhaustive enumeration performance of the FastKnock algorithm in a rich cultivation condition, we conducted additional in silico experiments considering succinate overproduction in Luri-Bertani (LB) medium. The *i*LB medium constraints were determined based on [64, 65].

The secondary metabolite, lycopene, as a heterologous product is produced in *E. coli* only under aerobic conditions. We considered two strains for lycopene production. For the first recombinant strain (*Strain1*), the lycopene biosynthesis pathway (i.e., the methylerythritol phosphate (MEP) pathway [66]) is added to the wild-type *E*. *coli* model [39, 67, 68]. For the second recombinant strain (*Strain2*), some other modifications are applied based on [69]. This provides an intracellular pool of pyruvate as the important precursor of lycopene production [70]. Additional file 2: Tables S1 and S2 in Supplement J.I show the maximum theoretical yield for the biosynthesis of the metabolites (i.e., maximum of *v*_*chemical*_) and our threshold for their production (*Th*_*chemical*_ = 0.05 $\times$* v*_*chemical*_).

Some results of the preprocessing phase is shown in Additional file 2: Table S3 of Supplement J.I, illustrating the number of reactions excluded from the search space before the main exploration procedure and before obtaining the removable reactions. The size of the search space is drastically reduced to 20% of all the reactions. In the *Reduced_model*, the blocked reactions and dead ends are removed [62]. Also, as described after the preprocessing phase, the search space is reduced iteratively and temporally during the search procedure of the FastKnock algorithm. This significantly reduces the number of linear programming problems (LPs) that must be solved. Specifically, compared to an exhaustive search, the reduction rates are 80%–85% for single knockouts, 96%–97.5% for double knockouts, 99.0–99.5% for triple knockouts, and above 99.8% for quadruple and quintuple knockouts (Table 1). The number of LPs is equal to the number of nodes in the traversal tree shown in Fig. 1, and it is independent of the target metabolite to be produced.

**Table 1:** The number of linear programming problems (LPs) solved by the FastKnock algorithm compared to an exhaustive search of the preprocessed search space (*Strain0* in *CM2* cultivation medium)

			Single	Double	Triple	Quadruple	Quintuple
*Strain0* in *CM2*	iJR904	Exhaustive search	208	21,528	1,478,256	75,760,620	3,091,033,296
		FastKnock	41	820	11,613	125,815	1,178,030
		**% Reduction**	**80.29**	**96.20**	**99.22**	**99.84**	**99.97**
	**iAF1260**	Exhaustive search	315	49,455	5,159,805	402,464,790	25,033,309,938
		FastKnock	57	1,506	25,985	348,966	4,058,061
		**% Reduction**	**81.91**	**96.96**	**99.50**	**99.92**	**99.99**
	**iJO1366**	Exhaustive search	385	73,920	9,437,120	901,244,960	68,674,865,952
		FastKnock	58	2,038	43,565	732,315	10,822,208
		**% Reduction**	**84.93**	**97.24**	**99.53**	**99.91**	**99.98**
	**iML1515**	Exhaustive search	403	81,003	10,827,401	1,082,740,100	86,402,659,980
		FastKnock	61	2193	58,750	1,674,010	25,489,714
		**% Reduction**	**84.87**	**97.30**	**99.46**	**99.85**	**99.98**

In comparison, in the exhaustive search the algorithm must check all the combinations of the reactions in the search space. For instance, iJR904 in *CM2* has 208 reactions in its search space. For finding double-knockout results in the exhaustive search, the algorithm must check all the double combinations of the elements in the search space (c(208, 2) = 21,528). Due to its time complexity, the exhaustive approach is not feasible for high-order reaction knockouts; thus, we compared FastKnock to a simple exhaustive search method for single, double, or triple knockouts. Our experiments showed that a significant reduction in the number of LPs is critical because it allows us to investigate and find all possible solutions.

Table 2 presents the total number of solutions obtained (regarding *CM2* cultivation medium) using the FastKnock algorithm. The results are reported in two cases: the maximum production rate (*v*_*max*_) and the guaranteed production rate (*v*_*grnt*_) as discussed in Supplement I.

**Table 2:** The number of solutions in iJR904 (*Strain0* in *CM2* cultivation medium)

Order of reaction knockout	*Strain0* in *CM2*					
	*succinate*		2-oxoglutarate		D-lactate	
	*v*_max_^*^	*v*_grnt_^**^	*v*_*max*_	*v*_*grnt*_	*v*_*max*_	*v*_*grnt*_
Single	2	1	0	0	0	0
Double	58	27	0	0	10	7
Triple	887	416	0	0	308	228
Quadruple	10,090	4794	0	0	4941	3790
Quintuple	98,300	48,693	29	0	58,481	13,639

^*^v_*max*_*: maximum production rate* (mmol.gDW^−1^ h^−1^)

^**^v_*grnt*_*: guaranteed production rate* (mmol.gDW^−1^ h^−1^)

We also compared our solutions to the results of the exhaustive search for single, double, and triple deletions for succinate production in iJR904 to verify the completeness of the FastKnock algorithm. Both approaches found two solutions for a single deletion. The exhaustive search for a double deletion found 398 solutions, of which only 58 solutions were true double deletions. The rest of the solutions were not acceptable because either (a) the combination of each single deletion solution and a zero-flux reaction was inappropriately considered as a double-deletion solution or (b) the elimination of a reaction in the co-knocked-out sets led to the removal of all the reactions in the set, while in the exhaustive search, the removal of each reaction in the set is counted as a separate solution. For triple deletions, the exhaustive search found 39,407 solutions, of which 887 were unique and acceptable. FastKnock found all the 887 solutions.

**Fig. 2:**
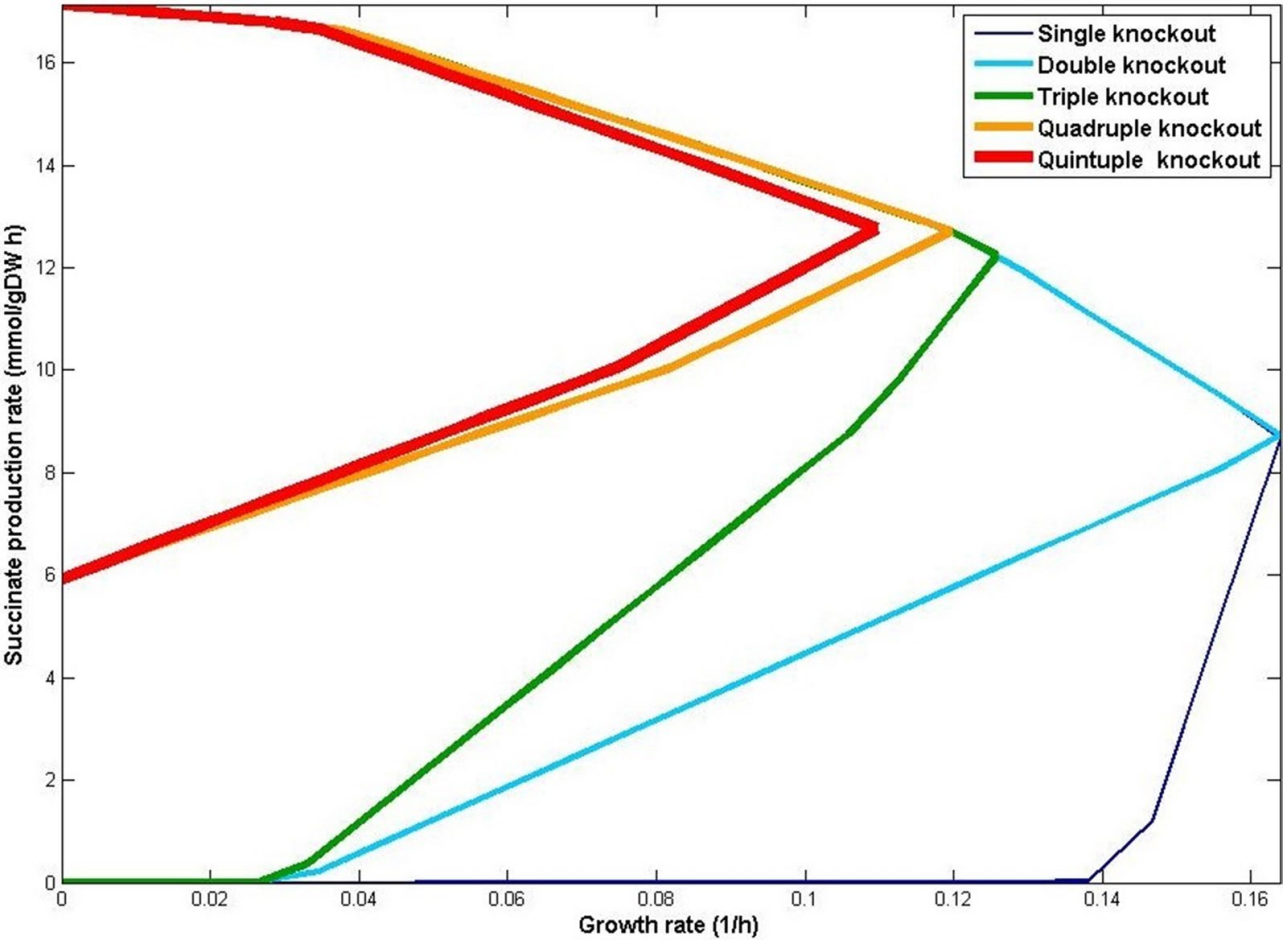
Production envelopes for the best solutions presented in Table 3 regarding succinate production from single to quintuple reaction deletions in iJR904. Knocking out more genes improves growth coupling. In particular, with quadruple and quintuple knockouts, significant production is guaranteed for any growth rate

Table 3 presents the best solutions found for iJR904 GEM (See also Additional file 2: Tables S4-S10). Supplement J.II includes the results for the iAF1260 (Additional file 2: Tables S11-S17) and iJO1366 (Additional file 2: Tables S18-S27) GEMs as well. As an example, we found that the best result for succinate overproduction is obtained by deleting one reaction, ADHEr, which is knocked out by the deletion of the gene b1241. Consequently, the deletion of the b1241 gene also causes the deletion of the LCADi_copy2 reaction. In this situation, the growth rate is 0.16 (h^−1^) as shown in the “biomass formation rate” column. After the deletion of ADHEr, the succinate production can vary between 5.11 and 9.50 mmol.gDW^−1^ h^−1^, which is more than the considered 0.85 mmol.gDW^−1^ h^−1^ threshold; hence, an acceptable amount of succinate production is guaranteed. Figure 2 presents the production envelopes calculated for the best cases presented in Table 3.

**Table 3:** The guaranteed rate of succinate growth-coupled production in in iJR904 (*Strain0* in *CM2* cultivation medium)

Number of knocked out reactions	Deleted reactions	Biomass formation rate (h^−1^)	Succinate production rate (mmol.gDW^−1^ h^−1^)		SoGC (h^−1^)	Deleted genes	Co-knockout reactions
			min	max			
Single	ADHEr	0.16	5.11	9.50	1.41E-2	b1241	LCADi_copy2
Double	ADHEr, LDH_D	0.15	8.08	9.51	1.43E-2	b1241, b2133, b1380	LCADi_copy2
Triple	ADHEr, LDH_D, PFL	0.12	11.08	12.73	1.53E-2	b1241, b2133, b1380, b3114, b0902, b3951	LCADi_copy, OBTFL
Quadruple	ADHEr, LDH_D, PFL, THD2	0.11	12.29	13.01	2.58E-2	b1241, b2133, b1380, b3114, b0902, b3951, b1602	LCADi_copy, OBTFL
Quintuple	ADHEr, LDH_D, GLUDy, PFL, THD2	0.10	12.34	13.06	2.61E-2	b1241, b2133, b1380, b1761, b3114, b0902, b3951, b1602	LCADi_copy, OBTFL

The analyses carried out with relatively older models, specifically iJR904, iAF1260, and iJO1366, were primarily focused on comparing the performance of FastKnock with both earlier methods (i.e., OptKnock) along with experimental studies and more recent approaches (i.e., MCSEnumerator) documented in the literature. As previously mentioned, additional tests were conducted to demonstrate that the effectiveness of the FastKnock method remains unaffected by the size of the model and the richness of the culture medium. These supplementary examinations included assessing succinate overproduction in medium *CM2* using model iML1515 and investigating succinate overproduction in *i*LB rich environment under aerobic conditions using both iJR904 and iML1515. The maximum rates of succinate growth-coupled production associated with these supplementary examinations are presented in Tables 4, 5, 6.

**Table 4:** The maximum rates of succinate growth-coupled production in iML1515 (*Strain0* in *CM2* cultivation medium)

Number of knocked out reactions	Deleted reactions	Biomass formation rate (h^−1^)	Succinate production rate (mmol.gDW^−1^ h^−1^)	Deleted genes	Co-Knockout reactions
Single	ATPS4rpp	0.25	12.73	b3735, b3737, b3738, b3732, b3733, b3736, b3734, b3731, b3739	–
Double	ATPS4rpp, PGL	0.24	16.54	b3735, b3737, b3738, b3732, b3733, b3736, b3734, b3731, b3739, b0767	–
Triple	PGI, ATPS4rpp, G6PDH2r	0.17	23.16	b4025, b3734, b3733, b3736, b3732, b3737, b3731, b3738, b3739, b3735, b1852	–
Quadruple	PFL, ACALD, THD2pp, THD2pp	0.19	23.49	b0351, b1241, b0903, b3951, b2579, b3952, b3114, b0902, b1602, b2913	OBTFL, 'ALCD2x', 'ALCD19'

**Table 5:** The maximum rates of succinate growth-coupled production in iJR904 in rich medium (*Strain0* in LB cultivation medium)

Number of knocked out reactions	Deleted reactions	Biomass formation rate (h^−1^)	Succinate production rate (mmol.gDW^−1^ h^−1^)	Deleted genes	Co-Knockout reactions
Single	ADHEr	1.35	20.10	b1241	LCADi_copy2
Double	F6PA, PFK	1.28	33.69	b0825, b3946, b3916, b1723	−
Triple	ACKr, GLCpts, PYK	0.56	54.88	b2296, b3115, b1849, b1819, b2415, b2416, b1621, b1101, b2417, b1817, b1818, b1854, b1676	DHAPT, GART, PPAKr
Quadruple	ACKr, ARGDC, GLCpts, PYK	0.56	64.72	b2296, b3115, b1849, b2938, b4117, b1819, b2415, b2416, b1621, b1101, b2417, b1817, b1818, b1854, b1676	GART, PPAKr, DHAPTs

**Table 6:** The maximum rates of succinate growth-coupled production in iML1515 in rich medium (*Strain0* in LB medium)

Number of knocked out reactions	Deleted reactions	Biomass formation rate (h^−1^)	Succinate production rate (mmol.gDW^−1^ h^−1^)	Deleted genes	Co-Knockout reactions
Single	ARGDC	1.08	19.72	b4117	–
Double	ARGDC, FADRx	1.05	22.09	b4117, b3844	FADRx, FE3Ri, FLVRx
Triple	NDPK5, ASPTA, ARGDC	1.03	28.14	b0474, b2518, b0928, b4054, b4117	ADK1, NDPK2, ADNK1, NDPK3, NDPK6, DADK, ADK4, NDPK1, ADK3, NDPK4, NDPK7, NDPK8, TYRTA, PHETA1, LEUTAi
Quadruple	NDPK5, PFL, LDH_D, ACALD	0.75	40.97	b0474, b2518, b2579, b3952, b0902, b3951, b0903, b3114, b1380, b0351, b1241	ADK1, NDPK2, ADNK1, NDPK3, NDPK6, DADK, ADK4, NDPK1, ADK3, NDPK4, NDPK7, NDPK8, OBTFL, ALCD2x, ALCD19

For practical applications, various evaluation indices, including product yield, SSP, and SoGC [55], and other important indices reflecting environmental and operational considerations, can be used to choose the most appropriate cases from the solutions found by FastKnock (Tables 7 and Table 8). In particular, the feasibility of CO_2_ biofixation and minimal production of undesired or toxic byproducts are also significant indexes for systems metabolic engineering purposes. For instance, an engineered strain that can simultaneously fix CO_2_ and produce a suitable biochemical might be preferred regarding environmental considerations. When all solutions are available, the analysis and identification of such appropriate cases is easily possible.

**Table 7:** The best solutions based on the desired evaluation indexes for succinate production under anaerobic condition (*Strain0* in *CM2* cultivation medium) in iJR904

Number of Knocked out reaction	Evaluation index											
	SSP **(**h^−1^**)** (FBA-based calculations)				SSP **(**h^−1^**)** (LinearMoMA-based calculations) [62])				SoGC **(**h^−1^**)** (FBA-based calculations)			
	Best knockout strategy	Biomass formation rate (h^−1^)	Succinate production rate (mmol g DW^−1^)	SSP (h^−1^) × 10	Best knockout strategy	Biomass formation rate (h^−1^)	Succinate production rate (mmol g DW^−1^)	SSP (h^−1^) × 10	Best knockout strategy	Biomass formation rate (h^−1^)	Succinate production rate (mmol g DW^−1^)	SSP (h^−1^) × 10
1	ADHEr	0.16	0.83	0.14	ADHEr	0.03	2.38	0.08	ADHEr	0.03	2.38	1.34
2	ADHEr, LDH_D	0.16	8.73	1.43	ADHEr, ATPS4r	0.12	8.32	1.01	ADHEr, LDH_D	0.16	8.73	1.35
3	ADHEr, LDH_D, PFL	0.12	12.24	1.53	ADHEr, ATPS4r, RPE	0.13	8.60	1.19	ADHEr, ATPS4r, LDH_D	0.16	8.90	3.01
4	ADHEr, LDH_D, PFL, URIK2	0.12	12.24	1.53	ADHEr, ATPS4r, LDH_D, RPE	0.13	8.71	1.20	ADHEr, LDH_D, HEX1, THD2	0.13	9.88	3.09
5	ADHEr, P, PFL, SUCOAS, RNDR3	0.12	12.25	1.54	ADHEr, ATPS4r, GLYK, F6PA, RPE	0.14	8.63	1.23	ADHEr, LDH_D, HEX1, THD2, DRPA	0.13	9.87	3.10

**Table 8:** Comparison of FastKnock, OptKnock and experimental results reported in the literature for succinate production. The iJR904 model (*Strain0*) is used in the in silico experimentations (M9 cultivation medium)

Knockout	Knockout strategy	Method	Biomass formation rate (h^−1^)	Succinate production rate (mmol.gDW^−1^ h^−1^)	yield	SSP (h^−1^) × 10	SoGC (h^−1^) × 100	CO_2_ exchange flux (mmol.gDW^−1^ h^−1^)
Triple	ADHEr, LDH_D, PTAr	OptKnock [33], FastKnock	0.08	9.37	0.94	0.75	0.79	**−9.36 (uptake)**
	ADHEr, LDH_D, PFL	OptKnock, FastKnock (best production rate)	0.12	**12.24**	**1.22**	**1.46E**	1.53	−5.87 (uptake)
	PTAr, PYK, GLCpts	OptKnock, FastKnock	0.09	9.32	0.93	0.83	0.87	3.24 (production)
	PFL, LDH_D, GLCpts	Experimental [71] (production is lower than considered threshold)	**0.16**	0.71	0.07	0.11	0.11	16.78 (production)
	ADHEr, ATPS4r, LDH_D	FastKnock (best SoGC)	**0.16**	8.90	0.89	1.42	**3.01**	−8.76 (uptake)
Quadruple	PTAr, PYK, ATPS4r, SUCD1i	OptKnock	**0.16**	1.18	0.11	0.18	0.01	9.03 (production)
	ADHEr, LDH_D, PFL, THD2	FastKnock (best production rate)	0.11	**12.72**	**1.27**	**1.39**	2.85	−6.12 (uptake)
	ADHEr, LDH_D, HEX1, THD2	FastKnock (best SoGC)	0.13	9.88	0.98	1.28	**3.09**	**−8.77** **(uptake)**
Quintuple	ADHEr, LDH_D, PTAr, PYK, GLCpts	OptKnock, FastKnock	0.05	9.96	0.99	0.49	1.19	**−9.51** **(uptake)**
	ADHEr, LDH_D, PFL, ACKr, FORt	Experimental [71], FastKnock	0.08	9.57	0.95	0.76	0.80	−9.16 (uptake)
	ADHEr, LDH_D, HEX1, THD2, DRPA	FastKnock (best SoGC)	**0.13**	9.87	0.98	**1.28**	**3.10**	−8.76 (uptake)
	ADHEr, LDH_D, GLUDy, PFL, THD2	FastKnock (best production rate)	0.10	**12.77**	**1.27**	1.27	2.61	−6.17 (uptake)

### Comparing FastKnock to OptKnock (case study: succinate overproduction in *E. coli* iJR904)

We analyzed FastKnock solutions in order to find the most appropriate outcomes based on three criteria, yield, SSP, and SoGC (Table 8). Additionally, the feasibility of CO_2_ biofixation is also examined and the relevant results are summarized, where a negative CO_2_ exchange flux represents a desirable CO_2_ uptake rate. We compared these best solutions obtained by FastKnock with the associated OptKnock results as well as experimental data available in the literature [71–73]. Note that OptKnock aims at, and terminates on, finding a single solution. Therefore, comparing it with FastKnock in terms of computational costs is not meaningful.

We found that a solution with the best production rate or an optimal solution of the optimization algorithms such as OptKnock does not necessarily bring the best SoGC and the other desired indexes. However, by identifying all the possible solutions for the problem, FastKnock allows a comprehensive analysis. For example, knocking out ADHEr, ATPS4r, and LDH_D is expected to lead to the best biomass formation rate (0.16 h^−1^) and the highest SoGC (3.01E-2 h^−1^), which is twice the best SoGC provided by OptKnock solutions while the other indices corresponding to this knockout are comparable with the best numbers shown in the table (i.e., a production rate of 8.90 vs. 12.24 mmol.gDW^−1^.h^−1^, a yield of 0.89 vs. 1.22, an SSP 1.42E-1 vs. 1.46E-1 h^−1^, and a CO_2_ exchange flux of −8.76 vs. −9.36 mmol.gDW^−1^.h^−1^). A relatively high value of SoGC can also be desirable from a dynamic perspective because it indicates that even under non-optimal conditions, the biosynthesis of the target biochemical is coupled with the growth of the production strain. This situation is usually encountered in batch and fed-batch cultivations in the logarithmic phase of growth.

A more striking example is the comparison between the PTAr, PYK, ATPS4r, and SUCD1i quadruple knockout identified by OptKnock with the two solutions with the best production rate (ADHEr, LDH_D, PFL, and THD2) and the best SoGC (ADHEr, LDH_D, HEX1, and THD2) identified by FastKnock. While the biomass formation rate of the FastKnock solutions (0.11, 0.13 h^−1^, respectively) are comparable with the OptKnock solution (0.16 h^−1^), the yield and SSP is an order of magnitude higher for FastKnock solutions. A serious issue with this OptKnock solution is the very low SoGC (1E-4 h^−1^), which indicates that the production rate would be hardly coupled with growth. In comparison, the predicted SoGC for FastKnock solutions are 2.85E-2 and 3.09E-2 h^−1^, respectively. Another disadvantage of OptKnock solution is a relatively high CO_2_ production rate of 9.03 mmol.gDW^−1^.h^−1^ while in the FastKnock solutions the CO_2_ exchange fluxes are −6.12 and −8.77 mmol.gDW^−1^.h^−1^, respectively.

Among the quintuple knockouts, the predicted SSP and SoGC for one of the FastKnock solutions (ADHEr, LDH_D, GLUDy, PFL, and THD2) are almost twice those of the OptKnock solution (ADHEr, LDH_D, PTAr, PYK, and GLCpts) while the other indices are comparable.

An important concern about OptKnock is possible false positive outcomes due to different scenarios. Firstly, false positives could be obtained due to the associated linear programming problem, focusing on maximizing the target reaction flux neglecting minimum possible production flux. In other words, OptKnock relies on FBA, potentially leading to false positives by not considering flux variabilities [43]. In contrast, FastKnock could guarantee the minimum production flux, regarding FVA. The second scenario is about the nature of the associated primal bi-level optimization problem, which is reformulated in the form of a single-level Mixed-Integer Linear Programming (MILP) problem. To solve the MILP problem, OptKnock utilizes the branch and bound method, which may generate false positives and even pose a risk of the algorithm getting trapped in an infinite loop. In contrast, FastKnock employs a different approach based on a search problem to explore the entire solution space. With appropriate evaluation criteria, unlike OptKnock, if it fails to provide a solution, it implies that no valid solution exists for the given criteria.

It is also important to note that, in some cases, false positives stem from limitations of the models due to incomplete knowledge of the genotype–phenotype relationships of the (micro)organism at hand in the process of model development. In this case, any in silico strain design approach intrinsically produces false positives [19].

### Comparing FastKnock to MCSEnumerator (case study: ethanol overproduction in *E. coli* iAF1260)

As mentioned previously, MCSEnumerator is a novel method for metabolic engineering based on the identification of minimal cut sets [50]. This approach applies a filtering step to reduce the computation time, which allows the user to find thousands (but not all) of the most efficient knockout strategies in genome-scale metabolic models. MCSEnumerator can be used to find a large number of metabolic engineering interventions, but it has various drawbacks. In this section, we compare MCSEnumerator with FastKnock. To aid in this comparison, we consider the case study of ethanol production in *E. coli* i*AF1260* GEM with an 18.5 mmol.gDW^−1^ h^−1^ glucose uptake rate under anaerobic conditions (*i*M9 medium) as presented in the MCSEnumerator publication.

**Fig. 3:**
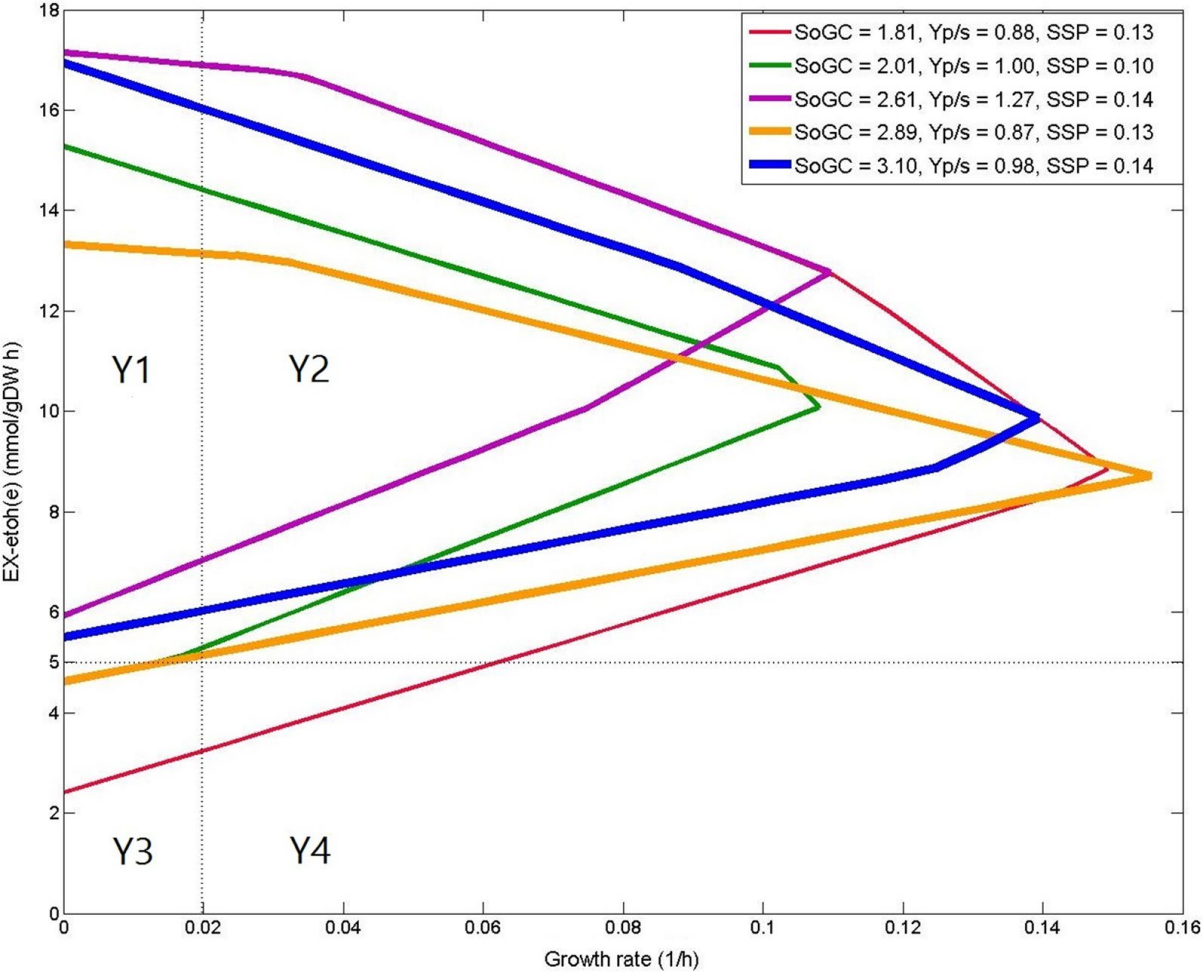
Five exemplar production envelopes for strategies identified by FastKnock for ethanol production in iAF1260, which is partitioned into four regions based on the growth rate (x axis) and the production flux (y axis) as in [15]. The horizontal dashed line indicates the threshold for production rate as considered in [15], and the vertical dashed line indicates the growth rate threshold. SoGC(× 100), product yield (Yp/s) and SSP(× 10) of the quadruple knockout strategies are shown in the top right legend. Unlike FastKnock, MCSEnumerator finds none of these strategies except the one shown in blue

We should discuss the effect of the MCSEnumerator thresholds on its solution set. It would not be feasible to apply MCSEnumerator using thresholds that are relaxed enough to find all the solutions (Supplement H). We illustrate this with an example in Fig. 3. The blue production envelope, which has the best SoGC value, is associated with a solution found by both MCSEnumerator and FastKnock. The associated solutions (with the red and green diagrams), which are the worst cases among the shown envelopes, were not found by MCSEnumerator because of the production threshold considered. This illustrates the efficiency of the primary filtration of the MCSEnumerator method. The starting point might not be the best factor for filtering appropriate solutions. For example, the minimum production rate based on the orange envelope is similar to the green envelope in Region Y3, which is below the threshold considered for ethanol production flux. Nevertheless, the orange envelope may still be associated with a proper solution due to its relatively high SoGC, but it was not found by MCSEnumerator.

Moreover, the predefined thresholds may result in the situation where some solutions obtained by MCSEnumerator are not necessarily and genuinely minimal. This implies that an appropriate solution with a cardinality of 'n' might exist but goes undiscovered, while it may appear in some higher-order solutions (> n) that include irrelevant additional reactions.

While the MCSEnumerator algorithm and its modified versions may exhibit shorter execution times, the number of solutions they can provide, given certain settings, constitutes only a very small percentage of the total potential solutions. Therefore, comparing the MCSEnumerator and FastKnock algorithms based solely on execution time is not rational, as these algorithms neither yield the same output nor pursue the same objective.

## Discussion

Overproduction of biochemicals of interest coupled with significant growth rates might be optimistic and may not always be easily achievable due to e.g., competing pathways in a metabolic network [43]. This can lead to weak coupling especially under suboptimal growth conditions. Alternatively, strong coupling requires that production must occur even without growth [14]. Specifically, product synthesis rate is said to be strongly coupled with biomass formation if the product yields of all steady-state flux vectors are equal to or larger than a predefined product yield threshold [15]. Accordingly, SoGC is defined as the square of the product yield per unit substrate divided by the slope of the lower edge of the production curve [55] (see Fig. 2).

SoGC is a non-linear objective function and thus OptKnock and most of the in silico strain design methods cannot be used to find knockouts with optimal SoGC. OptGene [37] is a heuristic approach that can be used to identify a single knockout strategy with optimal SoGC [55]. However, knocking out the single identified solution by OptGene may not be practically feasible e.g., due to the genes’ loci. Therefore, identification of all knockout strategies by FastKnock is desired and provides the expert experimentalists with the opportunity to choose from a short list of knockout strategies that are filtered for a relatively high SoGC, SSP, yield, etc. This shortlist can be investigated for advantageous solutions in terms of environmental considerations such as CO_2_ biofixation [71, 72], minimal production of undesired or toxic byproducts, practicality of knocking or silencing genes, etc. (Table 8) [6, 55, 73–75].

We proposed an efficient next-generation algorithm, FastKnock, which identifies all proper reaction or gene knockout strategies (with predefined maximum number of deletions) for the overproduction of a desired biochemical. We reached this goal by significantly pruning the search space without omitting any solutions. For example, in our experiments, FastKnock was required to explore only 1% of the search space in the pruned model when identifying all triple-knockout strategies. The rate of this reduction increases as more reactions are knocked out (e.g., about 0.1% for quadruple-knockout strategies and about 0.01% for quintuple-knockout strategies) (Table 1). This drastic reduction of the search space enables our novel FastKnock method to find the set of all possible solutions in a feasible time duration.

Finding the best and most suitable trade-off between cellular growth and the production of the desired biochemical is one of the key benefits of FastKnock results. Moreover, determining all possible solutions allows for the selection of the most appropriate strategy based on any desired evaluation index, including product yield, SSP, and SoGC (Tables 7 and 8). This is an important and useful feature of our search strategy, especially for practical applications [59].

We compared FastKnock to MCSEnumerator [50], which has been shown to find more efficient solutions than the MCS methods [76–78]. We found that the solutions identified by MCSEnumerator may not be minimal. Also, due to initial filtering, MCSEnumerator misses solutions that may be practically more appropriate than the best solutions it finds. In comparison, FastKnock identifies all minimal solutions, which can be mined later based on any desired criteria.

**Fig. 4:**
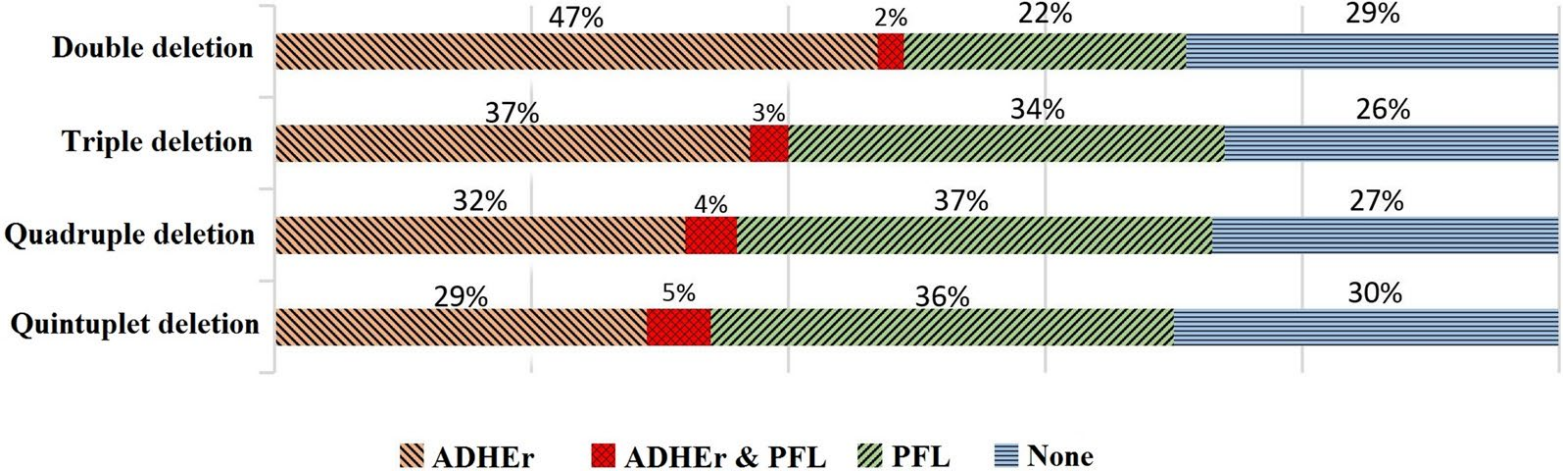
The rate of presence of the ADHEr and PFL reactions in all the possible solutions counted in Table 7 for succinate production

When all solutions are available, one interesting analysis that can be conducted is to identify the reactions or genes that are common among a relatively large number of solutions. For instance, in the case of iJR904, to produce succinate in *i*M9 under anaerobic conditions (*CM2*), about 70% of solutions include at least one of ADHEr or PFL reactions (Fig. 4). Moreover, when three or more reactions are to be deleted, the best results in terms of the succinate production rate include both ADHEr and PFL (Table 7). Collectively, this analysis suggests that ADHEr and PFL reactions support pathways that compete with succinate production, and these pathways are blocked when ADHEr and PFL are eliminated [79, 80]. Based on this analysis, we suggest using a heuristic for higher-level knockout combinations in which one or more reactions (e.g., ADHEr or PFL) are removed in searches for six or more knockouts. In this way, one would need to search for fewer reactions to knockout. We believe this heuristic would reduce the search space by an order of magnitude at the expense of losing not more than half of the solutions.

## Conclusion

While in silico strain design results do not necessarily lead to in vivo overproduction, obtaining all possible knockout strategies is critical for determining the best practical and most efficient strategy. The FastKnock algorithm is a general framework that can be used to overproduce any metabolite. It is not limited by factors such as richness and complexity of the cultivation conditions or large size of the metabolic network of the strain of interest. FastKnock identifies strategies, if exist, with a production rate higher than the desired threshold determined by the user.

### Supplementary Information

**Additional file 1: Supplement A**: Definitions and Overview of the Related Methods. Supplement B: The Optimization Methods. Supplement C: Preprocessing. Supplement D: FastKnock Dictionary. Supplement E: Co-Knockout Reactions. Supplement F: A Discussion about Finding all Knockout Strategies. Supplement G: Parallel implementation. Supplement H: MCSEnumerator Thresholds. Supplement I: Production Rate Guarantee.

**Additional file 2: Supplement J.I**: Model details and preprocessing results.

## Data Availability

All data generated or analyzed during this study are included in this published article [and its supplementary information files]. Our implementation of the FastKnock method in Python is publicly available at https://github.com/leilahsn/FastKnock.
