# Supplementary material for: FastKnock: an efficient next-generation approach to identify all knockout strategies for strain optimization
Source: Microb Cell Fact. 2024 Jan 29;23:37. doi: 10.1186/s12934-023-02277-x (PMC10823710; doi:10.1186/s12934-023-02277-x)
Supplement: Supplementary file 1 — Additional file 1: Supplement A: Definitions and Overview of the Related Methods. Supplement B: The Optimization Methods. Supplement C: Preprocessing. Supplement D: FastKnock Dictionary. Supplement E: Co-Knockout Reactions. Supplement F: A Discussion about Finding all Knockout Strategies. Supplement G: Parallel implementation. Supplement H: MCSEnumerator Thresholds. Supplement I: Production Rate Guarantee. [file 12934_2023_2277_MOESM1_ESM.docx]

# **Supplementary Materials**

## **Supplement A: Definitions and Overview of the Related Methods**

GEnome-scale metabolic Network REconstructions (GENREs) represent platforms organized biochemically, genetically, and genomically (BiGG) for target organisms, and these platforms contain their known metabolic reactions and the accompanying metabolic genes [1]. Organism-specific information about metabolic functionalities using gene-to-protein-to-reaction (GPR) associations [2] are included in GENREs, making them appropriately structured knowledge bases for analyzing genotype–phenotype relationships. Genome-scale models (GEMs), as chemically accurate mathematical formats of GENREs, are computational tools for the calculation or prediction of cellular states under different conditions [3][4].

The numerical matrix, S, as the key part of each GEM, characterizes the stoichiometry of the sum of all metabolic network reactions. In matrix S, each row represents a metabolite while each column signifies a reaction, and these elements represent the stoichiometric coefficients [5][6]. GEMs are considered the foundation of constraint-based models (CBMs), which are extensively utilized for quantitative analyses through COBRA (COnstraint-Based Reconstruction and Analysis) techniques [7][8][9]. In associated problems, constraints are mathematically considered as inequalities that represent bounds or equalities signifying balances. The rate of a metabolite transformation can be expressed as a reaction flux, for which an upper bound and a lower bound are considered based on the associated enzyme capacities as well as physicochemical and thermodynamic constraints [8][10][11].

The S matrix applies flux balance constraints on the network under steady-state conditions, thus guaranteeing that the total amount of each metabolite being generated is equal to the total amount being consumed. In the steady state, for each metabolite, a linear algebraic equation is derived that represents the balance of the relevant producing and consuming fluxes. Each feasible flux vector is calculated after pre-arranging all the input uptakes (to consider the cultivation medium) and imposing the condition-specific constraints. These vectors represent the functional states of the metabolic network (i.e., the reaction map).

The number of reactions, and consequently the number of reaction fluxes, in CBMs is typically more than the number of metabolites, and this leads to an undetermined system of equations. Therefore, optimization techniques are used to find plausible solutions. The principal objective of CBMs is considered to be the maximization of the flux through the biomass formation reaction (i.e., BOF: Biomass Objective Function). However, different objective functions may be considered depending on the definition of altered problems [8]. Flux balance analysis (FBA) [12] and flux variability analysis (FVA) [13] are popular COBRA optimization (biased) approaches for analyzing the flow of metabolites through a reaction map (see Supplement B). When applying biased COBRA approaches, a wide range of cellular functions or states can be predicted, including cellular growth capabilities in numerous media and the impact of gene knockouts on cellular physiology. Accordingly, various in silico metabolic engineering and strain design approaches have been developed to identify potential gene or gene reaction targets to be knocked out or up-/down-regulated for the growth-coupled production of biochemical(s) of interest [14][15].

Reactions have various functional roles in cellular metabolism. Based on these roles, different subsystems are considered in a metabolic network. A subsystem can represent any relationship between the functional roles, such as a set of enzymes that comprise a metabolic pathway [16]. Reactions can be clustered based on the main subsystems [17][18][19][20] or based on the associated metabolic genes. This latter approach improves the efficiency of the search procedure, as described in Supplement E.

There are two basic approaches for designing metabolic intervention strategies: top-down approaches (e.g., OptKnock [21], OptGene [25], MoMAKnock [22], CiED [26]) and bottom-up procedures (e.g., FSEOF [27], CosMos [28]) [29]. Several metaheuristic top-down algorithms have also been developed for in silico strain design. These algorithms can pinpoint the suboptimal solutions within a reasonable time.

Bottom-up approaches discover appropriate intervention strategies by comparing two flux distributions. One of these distributions relates to the wild-type, which mostly aims to maximize the cell’s growth rate. The other distribution relates to a specific functional state, which takes into account the goal of the desired biochemical production.

In a nutshell, top-down approaches use optimization methods to find an optimal solution at the cost of significant execution time. While top-down metaheuristic approaches require less computational resources, they are not guaranteed to find a globally optimal solution. On the other hand, bottom-up approaches can be used to find a set of solution candidates [14], but it is challenging to identify the proper solutions by comparing the flux distributions of the wild-type to the ideally engineered states.

FastKnock, as a next-generation knockout strategy algorithm, identifies all possible solutions for multiple reaction knockouts to overproduce a (bio)chemical of interest. Theoretically, an exhaustive search may find all possible solutions. Exhaustive search (aka complete search, brute force, or recursive backtracking) is a method for solving a problem by traversing the entire search space to find appropriate solutions. For example, to find triple knockout strategies by an exhaustive search, one would need to try all subsets of three knockouts. In contrast, FastKnock does not need to try all of these subsets. Instead, FastKnock uses the information that is available only during the search process to prune big portions of the rest of the search space. Specifically, at each node of the traversal tree, we check whether removing a certain reaction would lead to feasible solutions. If not, FastKnock prunes the whole subtree of that node. While this pruning step drastically reduces the search space, it cannot be done as a preprocessing step because the required information is not available until we visit that particular node of the traversal tree. During the search we can prune parts of the search space that we are sure do not lead to the required solution.

## **Supplement B: The Optimization Methods**

Various mathematical and optimization approaches are widely applied to analyze cellular metabolism--deploying CBMs. FBA and FVA are the most popular biased COBRA approaches based on linear programming [8][21]. Biased COBRA approaches involve optimizing an objective function to determine physiologically relevant flux distributions. This is due to the fact that a cell does not exploit the majority of potential functional states accessible in a metabolic network [8]. Equation (1) represents the optimization problem relevant to FBA. Assume that *S_ij_* represents the stoichiometric coefficient of the metabolite *i* in the reaction *j*, and *v_j_* is the flux value through reaction j that is in the range of *LB_j_* to *UB_j_*:

| $\begin{matrix} max \\ v \end{matrix}\begin{matrix} C^{T}v \\ \end{matrix}$ |  | (1) |
| --- | --- | --- |
| $\begin{matrix} subject to: \\ \end{matrix}$ | $\sum_{j} S_{ij}v_{j}=0, \forall i\in M, \forall j\in R$ ${LB}_{j}\leq v_{j}\leq{UB}_{j}$ , $\forall j\in R$ |

where *v* is a flux vector that contains the flux values of all the reactions in the model; *C* is the vector of the objective coefficients; *R* and *M* are the set of reactions and metabolites, respectively, present in the GEM. By solving this optimization problem, the fluxes of all the reactions in the cell are calculated under steady-state conditions for a predefined medium considering the relevant allowable exchange fluxes.

It should be noted that various solutions may exist for decision variables according to an optimal solution for the considered objective function. Decision variables in the FBA are the fluxes of all the reactions in the metabolic network. Obviously, for analyzing cellular metabolism, the objective function of the FBA problem could be the maximization of the flux of the biomass formation reaction (i.e., the cellular objective). The determined optimal value of the cellular objective is the maximum wild-type growth rate denoted by *gr_WT_*. FBA is also used to simulate the impact of genes or reactions knockout on the metabolic network functional state [22][23].

FVA determines the flux value ranges of all the reactions under a certain (sub)optimal condition using a two-step procedure. In the first step, FVA solves an optimization problem for determining the optimal value of the objective function (usually the cellular objective) in the same way as FBA. Then, two new optimization problems are defined for each reaction in the cell. The objective functions of these problems for the reaction *j* are shown in Equations:

| $v_{j, lower}= {min(v}_{j})$ |  | (2) |
| --- | --- | --- |

and

| $v_{j, upper}= {max(v}_{j})$ |  | (3) |
| --- | --- | --- |

The constraints on these problems are similar to the FBA constraints, except they have an additional constraint:

| ${gr}_{WT}\leq v_{biomass}\leq{gr}_{WT}$ |  | (4) |
| --- | --- | --- |

This means that the lower and upper bounds of the flux of the cellular objective reaction are both set to their optimal (or suboptimal as a percentage of optimal) value(s). The results of these two problems determine the permissible range of the flux of each reaction. It is not always applicable to consider the optimal solution for growth to determine the flux variability of the reactions. Conversely, the suboptimal growth conditions might have more proper flux distributions regarding metabolic engineering purposes. To analyze cellular metabolism under suboptimal cell growth, the FVA method might be used more efficiently. This FVA optimization problem is presented in Equation 5 [24] below:

| $\begin{matrix} max/min v_{j} \\ v \end{matrix}$ |  | (5) |
| --- | --- | --- |
| $\begin{matrix} subject to: \\ \end{matrix}$ | $\sum_{j} S_{ij}v_{j}=0, \forall i\in M, \forall j\in R$ $v_{biomass}\geq\gamma\times{gr}_{WT}$ ${LB}_{j}\leq v_{j}\leq{UB}_{j}$ , $\forall j\in R$ |

In Equation 5, *v_j,lower_* and *v_j,upper_* are the minimum and maximum flux values of reaction *j* that satisfy the problem constraints; hence, the flux of reaction j can vary in the range of [*v_j,lower_* , *v_j,upper_*]. The parameter $\gamma$ refers to the percentage of the growth in a particular suboptimal state ($0\leq\gamma\leq1$). Considering the parameter $\gamma= 1$ results in the optimal growth rate under steady-state conditions, and the constraint on the flux through the biomass is obviously *v_biomass_ = gr_WT_*. In the last constraint, *LB_i_* and *UB_i_* are the lower and upper bounds, respectively, for the flux of reaction *i*.

## **Supplement C: Preprocessing**

Preprocessing of the GEMs aims to reduce the search space before starting the main search algorithm. The preprocessing phase, as shown in Figure 5, consists of two main steps: one computational and one biological. The first step results in *a Reduced_model* that includes a subset of reactions from the original model.

| 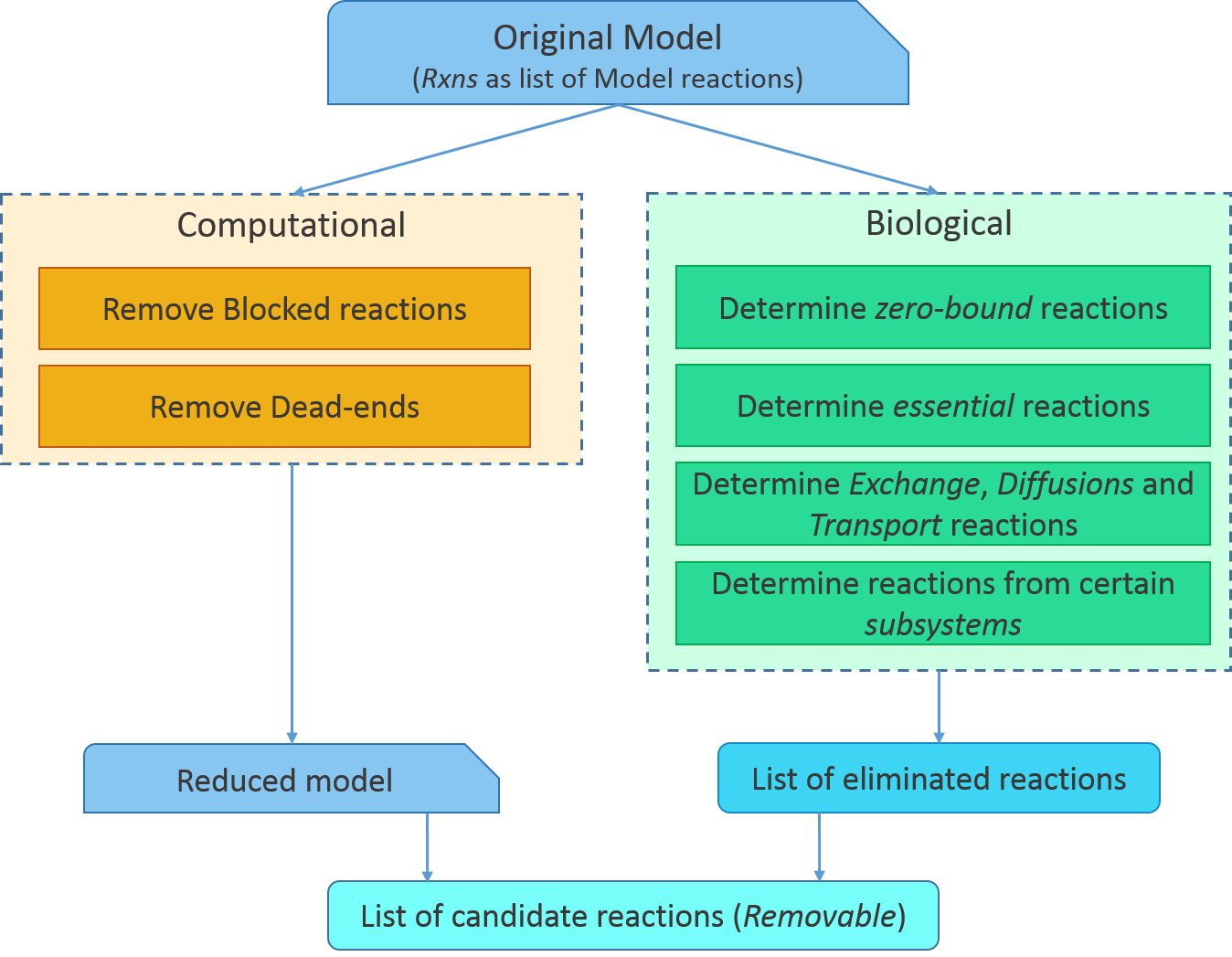 |
| --- |
| *Figure 5: Preprocessing includes computational and biological steps. The list of removable reactions in the metabolic model (indicated by “Removable”) is the result of the preprocessing phase.* |

The GEM of interest is prepared by imposing culture medium-associated constraints on the relevant GENRE (GENome-scale metabolic network REconstruction). Upon identifying the culture medium, some of the pathways in the cell are intuitively blocked. In a specific culture medium, the cell can consume certain amounts of some pre-specified compounds, hence no flux passes through certain pathways. All the reactions that belong to these pathways have zero flux. These *blocked* reactions are identified and removed in the first step of the preprocessing phase. Besides, given the incomplete knowledge of cellular genotype-phenotype relationships, there may exist some metabolites in the network that can structurally be consumed or produced by the corresponding reactions. Hence, they can block the flux of some reactions. These are termed dead ends. These reactions are also removed from the metabolic network. As mentioned in the manuscript, the primary search space (i.e., reactions of the original metabolic model called *Rxns*) is narrowed down in the preprocessing phase. The reactions in the set *Rxns* that do not exist in the *Reduced_model* (i.e., the *blocked* reactions and *dead ends*) are permanently removed from the metabolic model [25][26][27][28][29].

The second step of the preprocessing phase finds the reactions that are not appropriate candidates for deletion or should be excluded from the search space. These reactions are eliminated from the search space, but they should remain in the model. Several categories of reactions should be eliminated, including the *zero-bound*, *essential*, *exchanges*, *diffusions*, and *transport* reactions as well as the reactions that belong to certain subsystems (see Figure 5).

The *zero-bound* reactions in the metabolic network are those reactions that have both lower and upper flux bounds of zero. The existence of these reactions cannot affect the function of the network and can be eliminated from the search space. The *essential* reactions, which track the natural goal of a cell, are also eliminated from the search space. Computationally, by knocking out each of the *essential* reactions, the growth of the cell is reduced to typically less than 1% of the maximum *in silico* wild-type growth rate [30].

Since growth-coupled production is the goal of our proposed algorithm, the *essential* reactions are not proper candidates for deletion. Therefore, the *essential* reactions must be eliminated from the search space. In Supplement E, we demonstrate that eliminating these *essential* reactions from the search space does not affect the set of acceptable solutions (i.e., no acceptable solution is lost). Besides, the *transport* reactions that displace the metabolites in the cell cannot be appropriate choices for exclusion. Precisely, if it is beneficial to discontinue the production of a metabolite, the reaction that leads to its production must be knocked out rather than the reaction that displaces it.

In addition to the reactions responsible for the transport of metabolites across the cell membrane, the *exchange* and *diffusion* reactions cannot be easily removed biologically. Therefore, this category of reactions is eliminated from the search space. Inspired [28], the reactions from certain *subsystems* (see Supplement A) including *cell envelope biosynthesis*, *glycerophospholipid metabolism*, *inorganic ion transport and metabolism*, *lipopolysaccharide biosynthesis and recycling*, *membrane lipid metabolism*, *murein biosynthesis*, *murein recycling*, *inner membrane transport*, *outer membrane transport*, *outer membrane porin transport*, and *tRNA charging* are also eliminated from the search space. Obviously, by preprocessing, the reactions that belong to $Rxns-(Reduced_{model}.reactions \cap Removable)$ are not proper candidates for removal, and the deletion of these reactions has no structural effect on the metabolic network.

The result of the preprocessing phase is a list of candidate reactions for deletion from the model, called *Removable*. *Removable* is obtained by removing the reactions in the elimination list (obtained from the second step of preprocessing) from the set of *Reduced_model* reactions (obtained from the first step of preprocessing). This set of candidate reactions is considered the search space for the proposed search procedure.

## **Supplement D: FastKnock Dictionary**

| *Biomass reaction* | The reaction that simulates cellular reproduction. |
| --- | --- |
| *C(a, b)* | Combination of a and b = a! / (b!(a-b)!) |
| *target_level* | The number of desired reactions to be simultaneously deleted from the model. |
| *model* | The preprocessed and reduced metabolic model. This model is produced by removing blocked reactions and dead-ends from the original model in a specific medium culture. |
| *Removable* | The set of removable reactions in the model. |
| *queue_l_* | The nodes that must be investigated at level l of the traversal. |
| *checked_l_* | The set of all currently checked reactions in level l that should not be further investigated during level l. |
| *root* | the root node of the traversal tree. It contains all reactions after the preprocessing. |
| *Rxns^+^* | It is the list of reactions that have nonzero flux. |
| *Chemical* | The desired biochemical considered for growth-coupled overproduction. |
| *Co_knockedOut_r_* | The reactions that must be deleted along with *reaction* r based on the gene rules. |
| *gr_wt_* | The maximum wild-type growth rate. |
| *v_grnt_* | The guaranteed production rate. |
| *v_max_* | The maximum production rate. |
| *Solution_FBA* | The set of all maximized solutions. |
| *Solution_FVA* | The set of all guaranteed solutions. |
| *Th_chemical_* | The threshold for chemical production. |
| *v_chemical_* | The flux of the desired chemical. |
| *Node X* | A node in the traversal tree that represents a knockout strategy in the search procedure. |
| *Level* | Represents the depth of a node in the traversal tree. |
| *deleted_rxns* | List of deleted reactions for a node. |
| *target_space* | Target space of a node, that is the list of reactions. |
| *flux_dist* | Optimal flux distribution of the model in which all reactions of *deleted_rxns* are knocked out. |
| *model_X_* | The model obtained by removing the set *X.deleted_rxns* from the model. |
| *Target_Space_X_* | The set of the remaining reactions that could be an appropriate candidate for the next deletion after knocking out X from the model. |
| *Rxns_X_* | The set of all reactions in the metabolic model_X_. |
|  |  |
| *FluxDist_X_* | An optimal flux distribution of the reactions in the model_X_ using FBA. |

## **Supplement E: Co-Knockout Reactions**

The results of an *in-silico* simulation should be applicable to *in vivo* experiments. To accomplish this goal, the requirements of the problem must be as close to the real situation as possible. One of these requirements is to consider genes as the foundation of the reactions. It should be noted that in many research studies, the reactions are the basis of the search processes, and a set of reactions are reposted whose removal yields a significant overproduction in *in silico* simulation [8][31]. In fact, a single reaction could not be removed from a living cell while its genes are being manipulated *in vivo*. Therefore, the mapping of reactions to genes should be considered in the algorithm to reach realizable results. In other words, a reaction is knocked out from the network based on its associated gene rule.

The gene rule of each reaction is responsible for coding the requisite accompanying enzyme(s). In fact, the enzymes coded and controlled by the genes interact with the attendant metabolites to catalyze a biochemical reaction. A gene rule contains one or more genes as operands and the logical AND/OR relations as operators. In other words, the GEM elaborates on the gene rules associated with all its reactions.

For each reaction, we define an *involved set*, which represents its related genes. The involved set includes zero or more *essential sets*. The activation of at least one of the essential sets is sufficient for regulating a reaction. For activating an essential set, all the genes in the set must be triggered simultaneously. Based on this definition, to knock out a reaction, all the essential sets must be involved: We must eliminate at least one of the genes in each essential set.

*In vivo*, the removal of a reaction is performed by removing a set of genes. Generally, the removal of these genes affects not only the desired reaction but a set of reactions. Here, a novel approach is proposed to further reduce the search space. For each reaction *r*, based on the gene rules (i.e., the involved set and its essential sets), a (minimal) set of reactions named *Co_KnockedOut_r_* is determined. The set *Co_KnockedOut_r_* includes all reactions that are intrinsically removed by the deletion of the set of genes. It is noteworthy that for the reaction $s \in{Co\_KnockedOut}_{r}$, we cannot confirm whether *r* belongs to *Co_KnockedOut_s_*. For instance, let us suppose that only two reactions (*r* and *q*) exist with the following gene rules (GPR): $= g_{1}\&g_{2}, q= g_{1}$ , in which the AND operator is shown by “&.” The set *Co_KnockedOut_q_* is {*r*}, but the set *Co_KnockedOut_r_* is empty.

To determine the set *Co_KnockedOut_r_*, we first must identify the involved set and its essential sets of genes for *r*. To do this, we sort reactions into five categories:

1. **Reactions with unknown gene rules (orphans).** The gene rule for these reactions has not yet been discovered. For these *orphan* reactions, the involved set is empty (i.e., no essential gene is recognized).
2. **Reactions with single-gene gene rules.** These reactions have a single gene as the gene rule. This gene is essential for the reaction, and the involved set contains one essential set. For example:

$$r:g_{1} \to{involved}_{r}=\{\left\{ g_{1} \right\}\}$$

3. **Some genes ORed in the gene rule.** The gene rule of these reactions contains two or more genes with logical OR relationships. For removing such a reaction, all the genes in its gene rule must be deleted. Hence, each of the genes in the gene rule is placed in an individual essential set. For example:

$$r:g_{1}\left| g_{2} \right| g_{3} \to{involved}_{r}=\{\left\{ g_{1} \right\},\left\{ g_{2} \right\}, \left\{ g_{3} \right\}\}$$

4. **Some genes ANDed in the gene rule.** All the genes in its gene rule must be activated for catalyzing this type of reaction. Therefore, all the genes in the gene rule belong to an essential set, and the involved set has one essential set. Deleting this type of reaction is more complex than the previous types. It should be noted that our goal is to apply minimal modifications to the cellular metabolic network. Since these reactions are knocked out by deleting at least one gene from the essential set, choosing an appropriate candidate gene is a key step. In other words, selecting a gene that is essential for the least number of reactions is a more proper choice (i.e., selection a gene having minimum degree when dealing with ANDed gene rule). To clarify this situation, consider the gene rules of three reactions *r*, *q*, and *s* that are defined as follows:

| **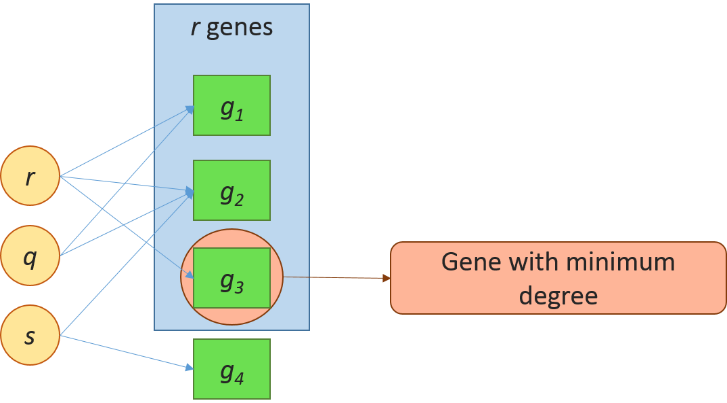** |
| --- |
| *Figure 1:* deleting reaction *r* that has ANDed gene-rule. Gene *g_1_*, *g_2_* or *g_3_* can be deleted for knocking out *r*. The best choice for deletion is *g_3_* since it is essential for the least number of reactions |

As shown in Figure 1, knocking out *g_3_* is the best choice for deleting *r* (i.e., it removes no other reactions), since the knockout of *g_1_* or *g_2_* leads to the deletion of *q* and *s*, respectively.

1. **Some genes ANDed/ORed in the gene rule.** For this type of reaction, the gene rules contain certain gene expressions that ORed with each other. Each expression has some ANDed genes. Therefore, the involved set contains more than one essential set. For example, consider the following gene rules of reactions *r*, *q*, and *s*:

| $r:\left( g_{1}\& g_{2} \right)\left\vert\left( g_{2}\& g_{3} \right) \right\vert\left( g_{4}\& g_{5} \right)$ | $\to$ | ${involved}_{r}=\left\{ \left\{ g_{1},g_{2} \right\},\left\{ g_{2},g_{3} \right\}, \left\{ g_{4},g_{5} \right\} \right\}$ |
| --- | --- | --- |
| $q:g_{4}$ | $\to$ | ${involved}_{q}=\left\{ \left\{ g_{4} \right\} \right\}$ |
| $s:\left( g_{2}\& g_{6} \right)$ | $\to$ | ${involved}_{s}=\left\{ \left\{ g_{2},g_{6} \right\} \right\}$ |

For deleting such a reaction, at least one gene from each essential set must be eliminated. Therefore, the deletion can be applied using various gene selections. Here as a simple heuristic, we select the genes that result in the minimum number of reaction deletions. Reaction *r* has three expressions in the gene rule:

$$Exp1:\left( g_{1}\& g_{2} \right)$$

$$Exp2:\left( g_{2}\& g_{3} \right)$$

$$Exp3:\left( g_{4}\& g_{5} \right)$$

For knocking out the reaction r, all of the expressions must be involved. Exp1 and Exp2 have a similar gene *g_2_*, so there are two methods for elimination: deleting {*g_1_*, *g_3_*} or deleting {*g_2_*}. A combination of these two sets by genes *g_4_* and *g_5_* provides different ways of deleting reaction *r*:

| $\{g_{1}, g_{3},g_{4}\}$ | $\to$ | $\{r,q\}$ |
| --- | --- | --- |
| $\{g_{1}, g_{3},g_{5}\}$ | $\to$ | $\{r\}$ |
| $\{g_{2},g_{4}\}$ | $\to$ | $\{r,s,q\}$ |
| $\{g_{2},g_{5}\}$ | $\to$ | $\{r,s\}$ |

After determining all sets, the set that knocks out the fewest reactions is chosen. This means that reaction *r* should be deleted by removing genes *g_1_*, *g_3_*, and *g_5_*.

We should note that in our experiments, only a small fraction of gene rule has complex gene rule relations. We have checked all of the reactions involved in these gene rules, one by one, to ensure not missing any solution.

In the preprocessing phase of the proposed framework, considering the above-mentioned categories and determining the involved sets and the essential sets for each reaction *r*, we can determine the *Co_KnockedOut_r_* set (i.e., the reactions that must be removed simultaneously along with r). It should be noted that the essential and transport reactions and their *Co_KnockedOut* reactions are excluded from the search space.

The presented method provides significant search space reduction, but it can be excluded from the main method with no loss of generality. This is an optional greedy heuristic preprocessing aiming at speedup and obtaining realistic results and generally speaking, a greedy approach may not necessarily lead to the best possible solution. On the other hand, this method can be incorporated as a preprocessing step in other metabolic engineering algorithms.

## **Supplement F: A Discussion about Finding all Knockout Strategies**

**Part 1:** As described in the preprocessing phase in Supplement C, the *essential* reactions are excluded from the search space before the main search procedure. In general, the flux distribution of the network may change by deleting each of the reactions. This may lead to the suspicion that combining the deletion of an essential reaction r with other reactions could be an acceptable solution for the new optimization problem that is obtained by deleting r. In this supplement, we show this cannot happen and prove that even by exploring the reduced search space, all feasible solutions will be found.

Removing a reaction *j* from the metabolic network corresponds to setting the lower and upper bounds of the flux of the corresponding reaction to zero, i.e., *LB_j_* = *UB_j_* = 0 in Equation (1) (Supplement B).

Naturally, the engineered cell should survive to produce the desired biochemical. Therefore, the growth rate, *μ*, should be greater than a certain cutoff (i.e., *μ_0_* > 0).

By definition, removing an essential reaction affects the flux range of the biomass and decreases the maximum value of the biomass flux to less than μ_0_ in the wild-type organism. Suppose that a new constraint *μ* > *μ_0_* is added to the problem. Hence, the feasible region will become empty (i.e., infeasible). According to the aforementioned note, any further changes in the network (i.e., removing a reaction or adding a constraint) cannot yield a feasible solution. Consequently, essential reactions are never candidates for removal. By excluding these essential reactions from the search procedure, the search space is significantly reduced and their exclusion does not affect the set of feasible solutions.

**Part 2:** In Section 2.1.2, we reduce the search space during the search procedure by temporarily ignoring the reactions that have a flux value of zero. In the optimization problem, each reaction *r* has an acceptable flux range [*minFlux_r_* , *maxFlux_r_*] which the value of flux must be in this range. This may lead to the suspicion that choosing another value in that range (except zero) for the reaction causes a different flux distribution in the network. Here, we show that no feasible solution would be missed due to this procedure.

Sensitivity analysis shows how changes in one variable in an optimization problem affect the optimal value of the objective function. In other words, if the value of the objective function is fixed, sensitivity analysis determines that each variable can be changed within a certain range. Choosing any value for the variable in the obtained range from sensitivity analysis will not change the optimal value of the objective function nor the allowable range of other variables. Therefore, if zero is within the allowable range of a variable, then the value of this variable can be considered to be zero without changing the optimal value of the objective function.

Based on the note mentioned above, knocking out a reaction with zero in its allowable flux range does not affect the value of the objective function nor the other flux ranges of the reactions. Therefore, in the FastKnock algorithm, at each level of reaction deletion, we exclude zero-flux reactions (i.e., reactions having zero in their flux ranges) from the search space.

## **Supplement G: Parallel implementation**

To further enhance the performance of the FastKnock process, we developed a parallel version of the FastKnock algorithm. This improvement decreases the execution time in proportion to the number of CPU cores. At each level *l*, each node in the *queue_l_* can be traversed independently from other nodes in that queue. Hence, the queue can be divided into multiple parts, which can be traversed in parallel because the traversed procedures are independent of each other. We developed a parallel version of the FastKnock algorithm based on dividing only the *queue_l_*. Based on the tree structure, the number of children of the nodes in each level follows a decreasing trend from beginning to the end. Therefore, in the task division between processors, *queue_1_* is divided into unequal parts. This means that we can easily generate processes up to the number of reactions present in the *queue_1_*. It should be noted that the FastKnock algorithm can also be parallelized in other levels.

## **Supplement H: MCSEnumerator Thresholds**

MCSEnumerator requires two thresholds: one threshold for the minimum biomass formation rate and another threshold for the minimum production rate. This latter threshold may omit some potentially appropriate solutions, especially when the evaluation criteria are nonlinear (e.g., production yield, SSP, SoGC) (Figure 3). Relaxing these thresholds does not solve this issue. This is due to the trade-off between the search speed and the results obtained from MCSEnumerator, both of which depend on the primary filtration thresholds. In contrast, FastKnock finds all possible solutions in a reasonable time frame, owing to its algorithmic capabilities and effective search space reduction. The search procedure and execution time of FastKnock are independent of any threshold or the growth-coupling overproduction of the desired biochemical(s). Moreover, MCSEnumerator must solve mixed integer linear programming (MILP) problems, while FastKnock uses linear programming (LP) problems, which have less computational complexity.

We compare the number of the results obtained from FastKnock to those obtained using MCSEnumerator, in the context of ethanol production in the *E.coli iAF1260* model. Neither method found any results for single deletions. However, these methods differed sharply in terms of the number of results obtained for multiple reaction deletions. FastKnock found 150 results for double deletions, while MCSEnumerator could not find any result. For triple and quadruple deletions, FastKnock found 7,738 and 342,328 solutions, respectively; while MCSEnumerator found only 2 and 98 solutions, respectively.

## **Supplement I: Production Rate Guarantee**

Maximizing the growth rate in the metabolic network using FBA leads to a certain flux range for each reaction (i.e., including the desired production rate(s) of biochemical(s)). Some previous studies simply assume that the maximized production rate would be achieved by maximizing the maximum flux (*maxFlux_r_*) of the chemical production reaction [32][33], ignoring the fact that the minimum flux (*minFlux_r_*) for the chemical production may be very small or even close to zero. To address this issue, other approaches (e.g., RobustKnock) [34] aimed to increase the minimum value of the desired biochemical production flux by applying max–min optimization. Hence, the production rate is guaranteed. Similarly, FastKnock uses FVA in the *identifyGuaranteedSolution* function to search for sets of reactions to knock out that increase the minimum production rate of the desired biochemical (Lines 5–8 of Algorithm 5).

Algorithm 5: Identifying guaranteed solution method

1: function ***identifyGuaranteedSolution*** (Node *X*, *model*, *biomass*, *chemical*, *Th_chemical_*, *FVA_solutions*)

2: **Input:**

*X*: a node of the tree,

*biomass*: a reaction that simulates the cellular growth rate

*chemical*: the desired chemical for growth coupling overproduction,

*Th_chemical_*: the threshold for *chemical* production rate,

*Fva_solutions*: the set of all guaranteed solutions

**Updates** *Fva_solutions*

3: **if** (*X.flux_dist* [*biomass*] > 0.01
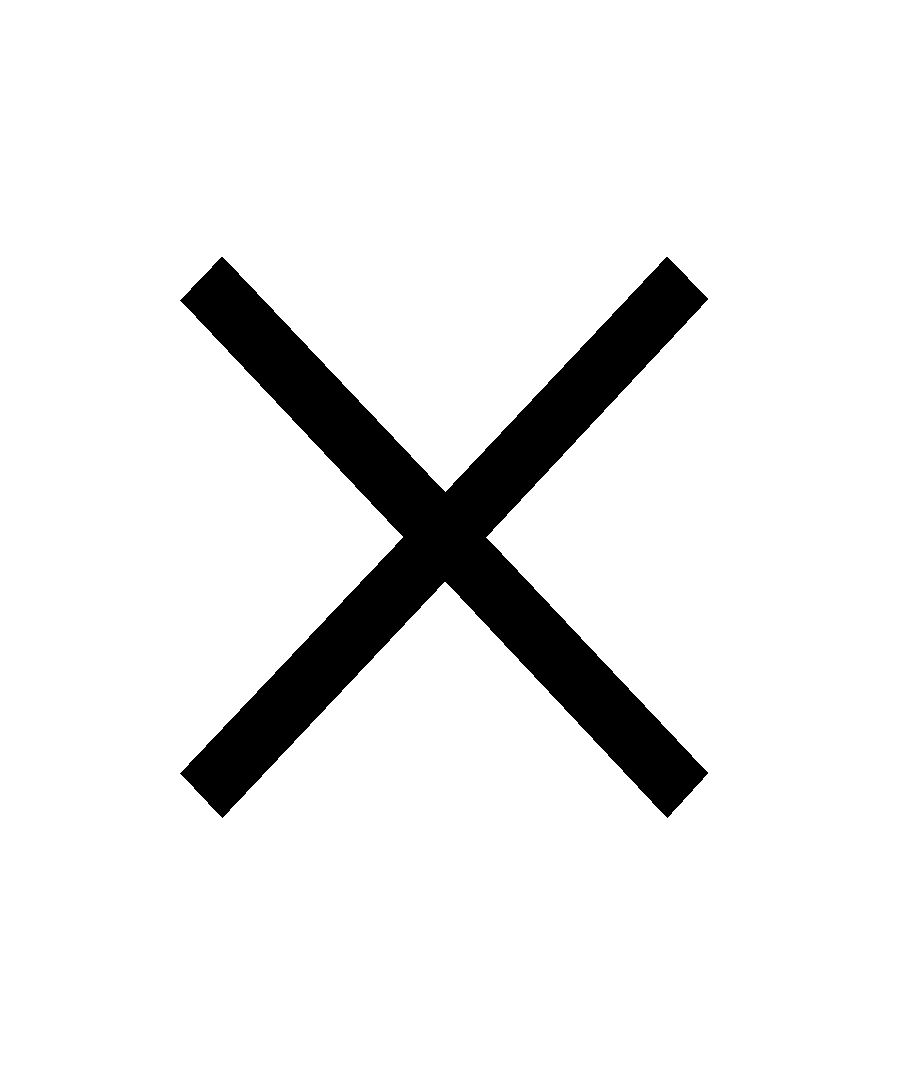
 *gr_wt_*) and (*X.flux_dist* [*chemical*] > *Th_chemical_*) **then**

4: **Construct** *model_X_* from model by setting the upper and lower bounds of all reactions in *X.deleted_rxns* to zero

5: *fva_result* = FVA (*model_X_*) ▷ FVA returns an optimal flux range for each reaction.

6: *min_production_value* = *fva_result* [*chemical*][minimum] ▷the lower bound of the *fva_result* flux range for the *chemical* reaction

7: if *min_production_value* > *Th_chemical_* **then**

8: **add** *X* to the *Fva_solutions*

# **References**

[1] M. A. Oberhardt, B. Ø. Palsson, and J. A. Papin, “Applications of genome-scale metabolic reconstructions,” *Mol. Syst. Biol.*, vol. 5, p. 320, Nov. 2009.

[2] I. Thiele and B. Ø. Palsson, “A protocol for generating a high-quality genome-scale metabolic reconstruction,” *Nat. Protoc.*, vol. 5, no. 1, pp. 93–121, 2010.

[3] E. J. O’Brien, J. M. Monk, and B. O. Palsson, “Using Genome-scale Models to Predict Biological Capabilities,” *Cell*, vol. 161, no. 5, pp. 971–987, May 2015.

[4] C. Gu, G. B. Kim, W. J. Kim, H. U. Kim, and S. Y. Lee, “Current status and applications of genome-scale metabolic models,” *Genome Biol.*, vol. 20, no. 1, p. 121, 2019.

[5] C. Zhang and Q. Hua, “Applications of Genome-Scale Metabolic Models in Biotechnology and Systems Medicine,” *Front. Physiol.*, vol. 6, p. 413, 2016.

[6] H. Kim, T. Y. Kim, and S. Y. Lee, “Metabolic flux analysis and metabolic engineering of microorganisms,” *Mol. Biosyst.*, vol. 4, pp. 113–120, Mar. 2008.

[7] B. Palsson, “Metabolic systems biology,” *FEBS Lett.*, vol. 583, no. 24, pp. 3900–3904, Dec. 2009.

[8] N. E. Lewis, H. Nagarajan, and B. O. Palsson, “Constraining the metabolic genotype-phenotype relationship using a phylogeny of in silico methods,” *Nat. Rev. Microbiol.*, vol. 10, no. 4, pp. 291–305, Feb. 2012.

[9] Y. Z. Lin Zeng, Qian-Yun Sun, Yang Jin, Yong Zhang, Wen-Hui Lee, “complement-depleting factor from king cobra, Ophiophagus hannah,” *sciencedirect*, vol. 60, no. 3, pp. 290–301, 2012.

[10] G. N. Stephanopoulos, A. a Aristidou, and J. Nielsen, “Metabolic Engineering: Principles and Methodologies,” *Metab. Eng.*, vol. 54, p. 725, 1998.

[11] M. Terzer, N. D. Maynard, M. W. Covert, and J. Stelling, “Genome-scale metabolic networks.,” *Wiley Interdiscip. Rev. Syst. Biol. Med.*, vol. 1, no. 3, pp. 285–297, 2009.

[12] J. D. Orth, I. Thiele, and B. O. Palsson, “What is flux balance analysis?,” *Nat. Biotechnol.*, vol. 28, no. 3, pp. 245–248, Mar. 2010.

[13] R. Mahadevan and C. H. Schilling, “The effects of alternate optimal solutions in constraint-based genome-scale metabolic models,” *Metab. Eng.*, vol. 5, no. 4, pp. 264–276, 2003.

[14] A. von Kamp and S. Klamt, “Growth-coupled overproduction is feasible for almost all metabolites in five major production organisms,” *Nat. Commun.*, vol. 8, p. 15956, Jun. 2017.

[15] J. Wang, R. Zhang, Y. Zhang, Y. Yang, Y. Lin, and Y. Yan, “Developing a pyruvate-driven metabolic scenario for growth-coupled microbial production.,” *Metab. Eng.*, vol. 55, pp. 191–200, Sep. 2019.

[16] M. DeJongh, K. Formsma, P. Boillot, J. Gould, M. Rycenga, and A. Best, “Toward the automated generation of genome-scale metabolic networks in the SEED,” *BMC Bioinformatics*, vol. 8, pp. 1–17, 2007.

[17] A. Wagner and D. A. Fell, “The small world inside large metabolic networks,” *Proc. R. Soc. B Biol. Sci.*, vol. 268, no. 1478, pp. 1803–1810, 2001.

[18] H. W. Ma, X. M. Zhao, Y. J. Yuan, and A. P. Zeng, “Decomposition of metabolic network into functional modules based on the global connectivity structure of reaction graph,” *Bioinformatics*, vol. 20, no. 12, pp. 1870–1876, 2004.

[19] S. M. Kelk, B. G. Olivier, L. Stougie, and F. J. Bruggeman, “Optimal flux spaces of genome-scale stoichiometric models are determined by a few subnetworks,” *Sci. Rep.*, vol. 2, p. 580, Aug. 2012.

[20] G. Basler, Z. Nikoloski, A. Larhlimi, A.-L. Barabási, and Y.-Y. Liu, “Control of fluxes in metabolic networks,” *Genome Res.*, vol. 26, no. 7, pp. 956–968, Jul. 2016.

[21] J. Schellenberger *et al.*, “Quantitative prediction of cellular metabolism with constraint-based models: The COBRA Toolbox v2.0,” *Nat. Protoc.*, vol. 6, no. 9, pp. 1290–1307, 2011.

[22] S. Mutturi, “FOCuS: A metaheuristic algorithm for computing knockouts from genome-scale models for strain optimization,” *Mol. Biosyst.*, vol. 13, no. 7, pp. 1355–1363, 2017.

[23] A. Chowdhury, A. R. Zomorrodi, and C. D. Maranas, “Bilevel optimization techniques in computational strain design,” *Comput. Chem. Eng.*, vol. 72, pp. 363–372, 2015.

[24] N. J. Stanford, P. Millard, and N. Swainston, “RobOKoD: microbial strain design for (over)production of target compounds,” *Front. Cell Dev. Biol.*, vol. 3, no. March, pp. 1–12, 2015.

[25] M. Ponce-de-León, F. Montero, and J. Peretó, “Solving gap metabolites and blocked reactions in genome-scale models: application to the metabolic network of Blattabacterium cuenoti,” *BMC Syst. Biol.*, vol. 7, no. 1, p. 114, 2013.

[26] A. Larhlimi, L. David, J. Selbig, and A. Bockmayr, “F2C2: a fast tool for the computation of flux coupling in genome-scale metabolic networks,” *BMC Bioinformatics*, vol. 13, p. 57, Apr. 2012.

[27] Z. Hosseini and S.-A. Marashi, “Discovering missing reactions of metabolic networks by using gene co-expression data,” *Sci. Rep.*, vol. 7, p. 41774, Feb. 2017.

[28] A. M. Feist, D. C. Zielinski, J. D. Orth, J. Schellenberger, M. J. Herrgard, and B. Ø. Palsson, “Model-driven evaluation of the production potential for growth-coupled products of Escherichia coli,” *Metab. Eng.*, vol. 12, no. 3, pp. 173–186, 2010.

[29] B. D. Heavner and N. D. Price, “Comparative Analysis of Yeast Metabolic Network Models Highlights Progress, Opportunities for Metabolic Reconstruction,” *PLOS Comput. Biol.*, vol. 11, no. 11, p. e1004530, Nov. 2015.

[30] D. Deutscher, I. Meilijson, S. Schuster, and E. Ruppin, “Can single knockouts accurately single out gene functions?,” *BMC Syst. Biol.*, vol. 2, 2008.

[31] K. Shabestary and E. P. Hudson, “Computational metabolic engineering strategies for growth-coupled biofuel production by Synechocystis,” *Metab. Eng. Commun.*, vol. 3, pp. 216–226, 2016.

[32] A. P. Burgard, P. Pharkya, and C. D. Maranas, “OptKnock: A Bilevel Programming Framework for Identifying Gene Knockout Strategies for Microbial Strain Optimization,” *Biotechnol. Bioeng.*, vol. 84, no. 6, pp. 647–657, 2003.

[33] P. Pharkya, A. P. Burgard, and C. D. Maranas, “OptStrain: A computational framework for redesign of microbial production systems,” *Genome Res.*, vol. 14, no. 11, pp. 2367–2376, 2004.

[34] N. Tepper and T. Shlomi, “Predicting metabolic engineering knockout strategies for chemical production: Accounting for competing pathways,” *Bioinformatics*, vol. 26, no. 4, pp. 536–543, 2009.
