# Supplementary material for: FastKnock: an efficient next-generation approach to identify all knockout strategies for strain optimization
Source: Microb Cell Fact. 2024 Jan 29;23:37. doi: 10.1186/s12934-023-02277-x (PMC10823710; doi:10.1186/s12934-023-02277-x)
Supplement: Supplementary file 2 — Additional file 2: Supplement J.I: Model details and preprocessing results. [file 12934_2023_2277_MOESM2_ESM.docx]

# Additional file

## **Supplement J. Results**

### **Supplement J.I: Model details and preprocessing results**

Table S1: The maximum capacity of Strain0 for the production of the primary metabolites of interest in CM1 and CM2 cultivation media

|  | ***iJR904*** | | | | ***iAF1260*** | | | | ***iJO1366*** | | | |
| --- | --- | --- | --- | --- | --- | --- | --- | --- | --- | --- | --- | --- |
|  | ***Strain0 in CM1*** | | ***Strain0 in CM2*** | | ***Strain0 in CM1*** | | ***Strain0 in CM2*** | | ***Strain0 in CM1*** | | ***Strain0 in CM2*** | |
|  | ***v_ch_^*^*** | ***Th_ch_^**^*** | ***v_ch_*** | ***Th_ch_*** | ***v_ch_*** | ***Th_ch_*** | ***v_ch_*** | ***Th_ch_*** | ***v_ch_*** | ***Th_ch_*** | ***v_ch_*** | ***Th_ch_*** |
| ***succ^+^*** | 17.14 | 0.85 | 17.14 | 0.85 | 16.72 | 0.83 | 14.89 | 0.74 | 17.09 | 0.85 | 16.89 | 0.84 |
| ***akg^++^*** | 12.00 | 0.60 | 4.93 | 0.24 | 12.69 | 0.63 | 5.12 | 0.25 | 12.89 | 0.64 | 6.31 | 0.31 |
| ***lac^+++^*** | 20.00 | 1.00 | 20.00 | 1.00 | 20.00 | 1.00 | 20.00 | 1.00 | 20.00 | 1.00 | 20.00 | 1.00 |

** v_ch_ : v_chemical_* (mmol.gDW^-1^h^-1^)

*** Th_ch_ : Th_chemical_* (mmol.gDW^-1^h^-1^)

*+ succ : succinate*

*++ akg : 2-oxoglutarate*

*+++ lac : D-lactate*

Table S2: The maximum capacities of Strain1 and Strain2 for the heterologous production of lycopene

|  | ***iJR904*** | | | | ***iAF1260*** | | | | ***iJO1366*** | | | |
| --- | --- | --- | --- | --- | --- | --- | --- | --- | --- | --- | --- | --- |
|  | ***Strain1 in CM1*** | | ***Strain2 in CM1*** | | ***Strain1 in CM1*** | | ***Strain2 in CM1*** | | ***Strain1 in CM1*** | | ***Strain2 in CM1*** | |
|  | ***v_ch_*** | ***Th_ch_*** | ***v_ch_*** | ***Th_ch_*** | ***v_ch_*** | ***Th_ch_*** | ***v_ch_*** | ***Th_ch_*** | ***v_ch_*** | ***Th_ch_*** | ***v_ch_*** | ***Th_ch_*** |
| ***lyc^*^*** | 0.865 | 0.04 | 1.01 | 0.05 | 0.865 | 0.04 | 1.02 | 0.05 | 1.01 | 0.05 | 1.02 | 0.05 |

* lyc : lycopene

Table S3: The number of different types of reactions and the statistics of the preprocessing phase (Strian0)

|  | ***iJR904*** | | ***iAF1260*** | | ***iJO1366*** | |
| --- | --- | --- | --- | --- | --- | --- |
| **Reaction** | **1075** | | **2382** | | **2583** | |
| **Step 1 of preprocessing** | | | | | | |
|  | ***Strain0 in CM1*** | ***Strain0 in CM2*** | ***Strain0 in CM1*** | ***Strain0 in CM2*** | ***Strain0 in CM1*** | ***Strain0 in CM2*** |
| Reduced model reactions | 667 | 633 | 1532 | 1470 | 1705 | 1657 |
| **Step 2 of preprocessing** | | | | | | |
| Zero-bound reactions | 0 | | 4 | | 10 | |
| Essential reactions | 270 | | 465 | | 509 | |
| Exchange reactions | 143 | | 299 | | 324 | |
| Reactions belong to special subsystems | 302 | | 1255 | | 1355 | |
| **Eliminated reactions** | **641** | | **1782** | | **1930** | |
| **Preprocessing result** | | | | | | |
| ***Removable reactions*** | **231** | **208** | **352** | **315** | **415** | **385** |

### **Supplement J.II: FastKnock results**

*Table S4: The number of solutions in iJR904 (CM1 cultivation medium)*

| Order of reaction knockout | ***Strain0 in CM1*** | | | | | | ***Strain1 in CM1*** | | ***Strain2 in CM1*** | |
| --- | --- | --- | --- | --- | --- | --- | --- | --- | --- | --- |
|  | **succinate** | | **2-oxoglutarate** | | **D-lactate** | | **lycopene** | | **lycopene** | |
|  | ***v_max_^*^*** | ***v_grnt_^**^*** | ***v_max_*** | ***v_grnt_*** | ***v_max_*** | ***v_grnt_*** | ***v_max_*** | ***v_grnt_*** | ***v_max_*** | ***v_grnt_*** |
| **Single** | 1 | 0 | 0 | 0 | 0 | 0 | 0 | 0 | 0 | 0 |
| **Double** | 42 | 0 | 0 | 0 | 2 | 0 | 0 | 0 | 0 | 0 |
| **Triple** | 933 | 10 | 0 | 0 | 76 | 0 | 0 | 0 | 0 | 0 |
| **Quadruple** | 15904 | 505 | 0 | 0 | 1605 | 8 | 0 | 0 | 4 | 0 |
| **Quintuple** | 213768 | 11827 | 380 | 0 | 23297 | 333 | 0 | 0 | 154 | 4 |

** v_max_ : maximum production rate* (mmol.gDW^-1^h^-1^)

** *v_grnt_ : guaranteed production rate* (mmol.gDW^-1^h^-1^)

Table S5: The maximum rates of the primary metabolites growth-coupled production in iJR904 (Strain0 in CM1 cultivation medium)

| **Chemical** | **Number of knocked out reactions** | **Deleted reactions** | ***μ* (*h^-1^*)** | ***v_chemical_*** (mmol.gDW^-1^h^-1^) | **SSP^*^ (*h^-1^*)** | **Deleted genes** | **Co-Knockout reactions** | |
| --- | --- | --- | --- | --- | --- | --- | --- | --- |
|  |  |  |  |  |  |  | **In the reduced model** | **Not in the reduced model** |
| **succ** | **1** | FUM | 0.76 | 0.79 | 0.0609 | b1612, b1611, b4122 | - | - |
|  | **2** | FUM, PTAr | 0.71 | 3.66 | 0.2620 | b1612, b1611, b4122, b2297, b2458 | PTA2 | - |
|  | **3** | GHMT2r, GND, SUCD1 | 0.53 | 6.25 | 0.3325 | b2551, b2029, b0722 | ALATA_D2, ALATA_L2, SUCD4 | - |
|  | **4** | GHMT2r, GND, MALS, SUCD1 | 0.53 | 6.26 | 0.3326 | b2551, b2029, b4014, b2976, b0722 | ALATA_D2, ALATA_L2, SUCD4 | - |
|  | **5** | ATPS4r, F6PA, PFL, PTAr, PYK | 0.13 | 8.84 | 0.1189 | b3739, b0825, b3946, b3114, b0902, b3951, b2297, b2458, b1854, b1676, | OBTFL, PTA2 | - |
| **akg** | **5** | ACKr, AKGDH, ATPS4r, THRS, G5SD | 0.17 | 3.99 | 0.0697 | b2296, b3115, b1849, b0727, b3739, b0004, b0243, | PPAKr, GART | 4HTHRS |
| **lac** | **2** | ADHEr, PTAr | 0.73 | 3.97 | 0.2928 | b1241, b2297, b2458 | LCADi_copy, PTA2 | - |
|  | **3** | NADH6, PFL, PDH | 0.38 | 9.43 | 0.3617 | b2285, b0114, b3951 b3114, b0902 | NADH7, NADH8, OBTFL | - |
|  | **4** | ATPS4r, HYD3, PFL, PDH | 0.15 | 12.69 | 0.2012 | b3739, b0974, b2994, b3951, b3114, b0902, b0114 | HYD2, OBTFL | - |
|  | **5** | ATPS4r, CBMKr, GLYCDx, PFL, PDH | 0.15 | 12.73 | 0.1987 | b3739, b0521, b0323, b2874, b3945, b3951, b3114, b0902, b0114 | OBTFL | - |

* SSP: substrate-specific productivity

Table S6: The maximum rate of heterologous lycopene growth-coupled production in iJR904 (Strain2 in CM1 cultivation medium)

| **Number of knocked out reactions** | **Deleted reactions** | ***μ* (*h^-1^*)** | ***v_chemical_*** (mmol.gDW^-1^h^-1^) | **SSP (*h^-1^*)** | **Deleted genes** | **Co-Knockout reactions** | |
| --- | --- | --- | --- | --- | --- | --- | --- |
|  |  |  |  |  |  | **In the reduced model** | **Not in the reduced model** |
| **5** | DRPA, GHMT2r, PGI, EDA, GLCDe | 0.47 | 0.41 | 0.0198 | b4381, b2551, b4025, b1850, b0124 | ALATA_L2, ALATA_D2 | - |

*Table S7: The maximum rates of the primary metabolites growth-coupled production* *in iJR904* (*Strain0 in CM2 cultivation medium*)

| **Chemical** | **Number of knocked out reactions** | **Deleted reactions** | ***μ* (*h^-1^*)** | ***v_chemical_*** (mmol.gDW^-1^h^-1^) | **SSP (*h^-1^*)** | **Deleted genes** | **Co-Knockout reactions** | |
| --- | --- | --- | --- | --- | --- | --- | --- | --- |
|  |  |  |  |  |  |  | **In the reduced model** | **Not in the reduced model** |
| **succ** | **1** | ADHEr | 0.16 | 8.64 | 0.1417 | b1241 | LCADi_copy2 | - |
|  | **2** | ADHEr, GLUDy | 0.14 | 8.76 | 0.1312 | b1241, b1761 | LCADi_copy2 | - |
|  | **3** | ADHEr, LDH_D, PFL | 0.12 | 12.24 | 0.1539 | b1241, b2133, b1380, b3951, b3114, b0902 | LCADi_copy2OBTFL | LDH_D2 |
|  | **4** | ADHEr, LDH_D, PFL, THD2 | 0.11 | 12.72 | 0.1522 | b1241, b2133, b1380, b1602, b3951, b3114, b0902 | LCADi_copy2OBTFL | LDH_D2 |
|  | **5** | ADHEr, LDH_D, GLUDy, PFL, THD2 | 0.10 | 12.77 | 0.1400 | b1241, b2133, b1380, b1761, b1602, b3951, b3114, b0902 | LCADi_copy2OBTFL | LDH_D2 |
| **akg** | **5** | THD2, PGL, ME2, GLUDC, AKGDH | 0.14 | 2.24 | 0.0327 | b1602, b2463, b3517, b1493, b0726 | - | - |
| **lac** | **2** | PFL, PDH | 0.13 | 18.00 | 0.2405 | b3951, b3114, b0902, b0114 | OBTFL | - |
|  | **3** | GLUDy, PFL, PDH | 0.12 | 18.16 | 0.2227 | b1761, b3951, b3114, b0902, b0114 | OBTFL | - |
|  | **4** | ADHEr, ATPS4r, MDH, PGL | 0.10 | 18.29 | 0.1893 | b1241, b3739, b3236 | LACDi_copy2 | - |
|  | **5** | ADHEr, ATPS4r, MDH, EDA, NADH8 | 0.10 | 18.42 | 0.1906 | b1241, b3739, b3236, b1850, b2285 | LACDi_copy2NADH7 | NADH6 |

Table S8: The guaranteed rates of the primary metabolites growth-coupled production in iJR904 (Strain0 in CM1 cultivation medium)

| **Chemical** | **Number of knocked out reactions** | **Deleted reactions** | ***μ* (*h^-1^*)** | ***v_chemical_*** (mmol.gDW^-1^h^-1^) | | **Deleted genes** | **Co-Knockout reactions** | |
| --- | --- | --- | --- | --- | --- | --- | --- | --- |
|  |  |  |  | ***min*** | ***max*** |  | **In the reduced model** | **Not in the reduced model** |
| **succ** | **3** | GND, MTHFC_1, SUCD1 | 0.54 | 5.30 | 6.11 | b2029, b0529, b0722 | MTHFD_1, SUCD4 | - |
|  | **4** | GHMT2r, GND, MALS, SUCD1 | 0.49 | 5.45 | 6.59 | b2551, b2029, b4014, b2976, b0722 | ALATA_D2, ALATA_L2, SUCD4 | - |
|  | **5** | ATPS4r, F6PA, PTAr, PYK, SUCD1 | 0.15 | 7.34 | 9.36 | b3739, b0825, b3946, b2297, b2458, b1854, b1676, b0722 | PTA2, SUCD4 | - |
| **lac** | **4** | ADHEr, ATPS4r, GLUDy, PTAr, | 0.22 | 6.85 | 11.45 | b1241, b3739, b1761, b2297, b2458 | LCADi_copy2 PTA2 | - |
|  | **5** | ACKr, ADHEr, ATPS4r, GLUDy, PGI | 0.13 | 10.36 | 13.09 | b2296, b3115, b1849, b1241, b3739, , b1761, b4025 | PPAKr, GART, LCADi_copy2 | - |

Table S9: The guaranteed rate of heterologous lycopene growth-coupled production in iJR904 (Strain2 in CM1)

| **Number of knocked out reactions** | **Deleted reactions** | ***μ* (*h^-1^*)** | ***v_chemical_*** (mmol.gDW^-1^h^-1^) | | **Deleted genes** | **Co-Knockout reactions** | |
| --- | --- | --- | --- | --- | --- | --- | --- |
|  |  |  | ***min*** | **max** |  | **In the reduced model** | **Not in the reduced model** |
| **5** | DRPA, GHMT2r, PGI, EDA, GLCDe | 0.47 | 0.12 | 0.47 | b4381, b2551, b4025, b1850, b0124 | ALATA_L2, ALATA_D2 | - |

Table S10: The guaranteed rates of the primary metabolites growth-coupled production in iJR904 (Strain0 in CM2 cultivation medium)

| **Chemical** | **Number of knocked out reactions** | **Deleted reactions** | ***μ* (*h^-1^*)** | ***v_chemical_*** (mmol.gDW^-1^h^-1^) | | **Deleted genes** | **Co-Knockout reactions** | |
| --- | --- | --- | --- | --- | --- | --- | --- | --- |
|  |  |  |  | ***min*** | ***max*** |  | **In the reduced model** | **Not in the reduced model** |
| **succ** | **1** | ADHEr | 0.16 | 5.11 | 9.50 | b1241 | LCADi_copy2 | - |
|  | **2** | ADHEr, LDH_D | 0.15 | 8.08 | 9.51 | b1241, b2133, b1380 | LCADi_copy2 | LDH_D2 |
|  | **3** | ADHEr, LDH_D, PFL | 0.12 | 11.08 | 12.73 | b1241, b2133, b1380, b3114, b0902, b3951 | LCADi_copy2 OBTFL | LDH_D2 |
|  | **4** | ADHEr, LDH_D, PFL, THD2 | 0.11 | 12.29 | 13.01 | b1241, b2133, b1380, b3114, b0902, b3951, b1602 | LCADi_copy2 OBTFL | LDH_D2 |
|  | **5** | ADHEr, LDH_D, GLUDy, PFL, THD2 | 0.10 | 12.34 | 13.06 | b1241, b2133, b1380, b1761, b3114, b0902, b3951, b1602 | LCADi_copy2 OBTFL | LDH_D2 |
| **lac** | **3** | PFL, NADH8, PDH | 0.13 | 16.78 | 18.11 | b3951, b3114, b0902, b2285, b0114 | NADH7, OBTFL | NADH6 |
|  | **4** | ADHEr, ATPS4r, MDH, ASPT | 0.12 | 17.52 | 18.30 | b1241, b3739, b3236, b4139 | LCADi_copy2 | - |
|  | **5** | ADHEr, ATPS4r, MDH, EDA, NADH8 | 0.10 | 17.81 | 18.53 | b1241, b3739, b3236, b1850, b2285 | LCADi_copy2 NADH7 | NADH6 |

Table S11: The number of solutions in iAF1260 (CM1 cultivation medium)

| Order of reaction knockout | ***Strain0 in CM1*** | | | | ***Strain1 in CM1*** | | ***Strain2 in CM1*** | |
| --- | --- | --- | --- | --- | --- | --- | --- | --- |
|  | **succ** | | **akg** | | **lyc** | | **lyc** | |
|  | ***v_max_^*^*** | ***v_grnt_^**^*** | ***v_max_*** | ***v_grnt_*** | ***v_max_*** | ***v_grnt_*** | ***v_max_*** | ***v_grnt_*** |
| **Single** | 0 | 0 | 0 | 0 | 0 | 0 | 0 | 0 |
| **Double** | 0 | 0 | 0 | 0 | 0 | 0 | 0 | 0 |
| **Triple** | 8 | 0 | 1 | 0 | 0 | 0 | 1 | 0 |
| **Quadruple** | 508 | 0 | 8 | 0 | 0 | 0 | 12 | 0 |
| **Quintuple** | 17120 | 263 | 370 | 0 | 0 | 0 | 580 | 0 |

** v_max_ : maximum production rate* (mmol.gDW^-1^h^-1^)

** *v_grnt_ : guaranteed production rate* (mmol.gDW^-1^h^-1^)

Table S12: The number of solutions in iAF1260 (CM2 cultivation medium)

| Order of reaction knockout | ***Strain0 in CM2*** | | | |
| --- | --- | --- | --- | --- |
|  | **succ** | | **akg** | |
|  | ***v_max_*** | ***v_grnt_*** | ***v_max_*** | ***v_grnt_*** |
| **Single** | 0 | 0 | 0 | 0 |
| **Double** | 0 | 0 | 0 | 0 |
| **Triple** | 0 | 0 | 0 | 0 |
| **Quadruple** | 125 | 0 | 0 | 0 |
| **Quintuple** | 8634 | 26 | 0 | 0 |

Table S13: The maximum rates of the primary metabolites growth-coupled production in iAF1260 (Strain0 in CM1 cultivation medium)

| **Chemical** | **Number of knocked out reactions** | **Deleted reactions** | ***μ* (*h^-1^*)** | ***v_chemical_*** (mmol.gDW^-1^h^-1^) | **SSP (*h^-1^*)** | **Deleted genes** | **Co-Knockout reactions** | |
| --- | --- | --- | --- | --- | --- | --- | --- | --- |
|  |  |  |  |  |  |  | **In the reduced model** | **Not in the reduced model** |
| **succ** | **3** | ACKr, ATPS4rpp, SUCDi | 0.13 | 5.84 | 0.0810 | b2296, b3115, b1849, b3737, b0722 | PPAKr, GART | - |
|  | **4** | ATPS4rpp, CYTBD2pp, FUM, PTAr | 0.13 | 6.02 | 0.0823 | b3737, b0979, b1612, b1611, b4122, b2297, b2458 | PTA2 | - |
|  | **5** | ACKr, ATPS4rpp, PFL, F6PA, PYK | 0.10 | 9.16 | 0.0970 | b2296, b3115, b1849, b3737, b3951, b0902, b0825, b3946, b1854, b1676 | PPAKr, GART', OBTFL | - |
| **akg** | **3** | ACKr, AKGDH, ATPS4rpp | 0.14 | 2.66 | 0.0383 | b2296, b3115, b1849, b0727, b3737 | PPAKr, GART | - |
|  | **4** | ACKr, ACALD, AKGDH, ATPS4rpp | 0.14 | 2.66 | 0.0383 | b2296, b3115, b1849, b1241, b0351, b0727, b3737 | PPAKr, GART | - |
|  | **5** | ACKr, ADPT, AKGDH, ATPS4rpp, PUNP1 | 0.14 | 4.55 | 0.0654 | b2296, b3115, b1849, b0469, b0727, b3737, b4384 | PPAKr, GART, PUNP2 | - |

Table S14: The maximum rates of succinate growth-coupled production in iAF1260 (Strain0 in CM2 cultivation medium)

| **Chemical** | **Number of knocked out reactions** | **Deleted reactions** | ***μ* (*h^-1^*)** | ***v_chemical_*** (mmol.gDW^-1^h^-1^) | **SSP (*h^-1^*)** | **Deleted genes** | **Co-Knockout reactions** | |
| --- | --- | --- | --- | --- | --- | --- | --- | --- |
|  |  |  |  |  |  |  | **In the reduced model** | **Not in the reduced model** |
| **succ** | **4** | F6PA, IMPD, PYK, ME2 | 0.10 | 7.61 | 0.0804 | b0825, b3946, b2508, b1854, b1676, b2463 | - | - |
|  | **5** | ACCOAL, F6PA, IMPD, PYK, RPE | 0.10 | 7.80 | 0.0814 | b0335, b0825, b3946, b2508, b1854, b1676, b3386, b4301 | - |  |

Table S15: The maximum rates of heterologous lycopene growth-coupled production in iAF1260 (Strain2 in CM1 cultivation medium)

| **Number of knocked out reactions** | **Deleted reactions** | ***μ* (*h^-1^*)** | ***v_chemical_*** (mmol.gDW^-1^h^-1^) | **SSP (*h^-1^*)** | **Deleted genes** | **Co-Knockout reactions** | |
| --- | --- | --- | --- | --- | --- | --- | --- |
|  |  |  |  |  |  | **In the reduced model** | **Not in the reduced model** |
| **4** | ALCD2x, PPM, THD2pp, LCARR | 0.70 | 0.098 | 0.0069 | b1478, b0356, b1241, b3380, b4383, b1602 | ALCD19, PPM2 | FALDH |
| **5** | ALCD2x, PSP_L, RNTR4c, THD2pp, MGSA | 0.57 | 0.31 | 0.0180 | b1478, b0356, b1241, b4388, b4238, b1602, b0963 | ALCD19, RNTR1c, RNTR2c, RNTR3c | FALDH |

Table S16: The guaranteed rate of succinate growth-coupled production in iAF1260 (Strain0 in CM1 cultivation medium)

| **Chemical** | **Number of knocked out reactions** | **Deleted reactions** | ***μ* (*h^-1^*)** | ***v_chemical_*** (mmol.gDW^-1^h^-1^) | | **Deleted genes** | **Co-Knockout reactions** | |
| --- | --- | --- | --- | --- | --- | --- | --- | --- |
|  |  |  |  | ***min*** | ***max*** |  | **In the reduced model** | **Not in the reduced model** |
| **succ** | **5** | ACKr, ATPS4rpp, PYK, F6PA, SUCDi | 0.12 | 7.51 | 9.65 | b2296, b3115, b1849, b3737, b1854, b1676, b0825, b3946, b0722 | PPAKr, GART | - |

Table S17: The guaranteed rate of succinate growth-coupled production in iAF1260 (Strain0 in CM2 cultivation medium)

| **Chemical** | **Number of knocked out reactions** | **Deleted reactions** | ***μ* (*h^-1^*)** | ***v_chemical_*** (mmol.gDW^-1^h^-1^) | | **Deleted genes** | **Co-Knockout reactions** | |
| --- | --- | --- | --- | --- | --- | --- | --- | --- |
|  |  |  |  | ***min*** | ***max*** |  | **In the reduced model** | **Not in the reduced model** |
| **succ** | **5** | PSP_L, THD2pp, PGL, ALDD2y, ME2 | 0.13 | 0.96 | 2.30 | b4388, b1602, b0767, b3588, b2463 | - | ALDD3y |

Table S18: Number of solutions in iJO1366 (CM1 cultivation medium)

| Order of reaction knockout | ***Strain0 in CM1*** | | | | ***Strain1 in CM1*** | | ***Strain2 in CM1*** | |
| --- | --- | --- | --- | --- | --- | --- | --- | --- |
|  | **succ** | | **akg** | | **lyc** | | **lyc** | |
|  | ***v_max_*** | ***v_grnt_*** | ***v_max_*** | ***v_grnt_*** | ***v_max_*** | ***v_grnt_*** | ***v_max_*** | ***v_grnt_*** |
| **Single** | 1 | 1 | 0 | 0 | 0 | 0 | 0 | 0 |
| **Double** | 72 | 68 | 0 | 0 | 0 | 0 | 3 | 2 |
| **Triple** | 1982 | 1968 | 0 | 0 | 0 | 0 | 191 | 129 |
| **Quadruple** | 842* | 297 | 13 | 0 | 0 | 0 | 6438 | 4818 |
| **Quintuple** | 15461** | 181 | 670 | 0 | 7 | 2 | 8706*** | 8704 |

* *Th_chemical_* = 1

** *Th_chemical_* = 3

*** *Th_chemical_* = 0.5

Table S19: Number of solutions in iJO1366 (CM2 cultivation medium)

| Order of reaction knockout | ***Strain0 in CM2*** | | | |
| --- | --- | --- | --- | --- |
|  | **succ** | | **akg** | |
|  | ***v_max_*** | ***v_grnt_*** | ***v_max_*** | ***v_grnt_*** |
| **Single** | 0 | 0 | 0 | 0 |
| **Double** | 1 | 0 | 0 | 0 |
| **Triple** | 61 | 7 | 0 | 0 |
| **Quadruple** | 1867 | 449 | 0 | 0 |
| **Quintuple** | 23751* | 13150 | 6 | 0 |

* *Th_chemical_* = 5

Table S20: The maximum rates of the primary metabolites growth-coupled production in iJO1366 (Strain0 in CM1 cultivation medium)

| **Chemical** | **Number of knocked out reactions** | **Deleted reactions** | ***μ* (*h^-1^*)** | ***v_chemical_*** (mmol.gDW^-1^h^-1^) | **SSP (*h^-1^*)** | **Deleted genes** | **Co-Knockout reactions** | |
| --- | --- | --- | --- | --- | --- | --- | --- | --- |
|  |  |  |  |  |  |  | **In the reduced model** | **Not in the reduced model** |
| **succ** | **1** | FUM | 0.85 | 0.89 | 0.0757 | b1612, b4122, b1611 | - | DTARTD |
|  | **2** | FUM, IDOND | 0.84 | 0.88 | 0.0747 | b1612, b4122, b1611, b4267 | IDOND2 | DTARTD |
|  | **3** | FUM, MTHFC, PFL | 0.76 | 1.56 | 0.1200 | b1612, b4122, b1611, b0529, b0902, b3951 | MTHFD, OBTFL | DTARTD |
|  | **4** | ACKr, FUM, GND, PGCD | 0.60 | 6.08 | 0.3687 | b3115, b2296, b1849, b1612, b4122, b1611, b2029, b2913 | PPAKr, GART | DTARTD |
|  | **5** | ATPS4rpp, F6PA, PFL, PTAr, PYK | 0.19 | 8.47 | 0.1632 | b3736, b0825, b3946, b0902, b3951, b2297, b2458, b1854, b1676 | PTA2 | - |
| **akg** | **4** | ACKr, AKGDH, ATPS4rpp, ABTA | 0.23 | 2.46 | 0.0569 | b3115, b2296, b1849, b0726, b3736, b2662, b1302 | PPAKr, GART | - |
|  | **5** | ACKr, ADPT, ALDD2y, ATPS4rpp, SUCOAS | 0.23 | 3.96 | 0.0922 | b3115, b2296, b1849, b0469, b3588, b3736, b0728 | PPAKr, GART, ALDD3y | - |

Table S21: The maximum rate of heterologous lycopene growth-coupled production in iJO1366 (Strain1 in CM1 cultivation medium)

| **Number of knocked out reactions** | **Deleted reactions** | ***μ* (*h^-1^*)** | ***v_chemical_*** (mmol.gDW^-1^h^-1^) | **SSP (*h^-1^*)** | **Deleted genes** | **Co-Knockout reactions** | |
| --- | --- | --- | --- | --- | --- | --- | --- |
|  |  |  |  |  |  | **In the reduced model** | **Not in the reduced model** |
| **5** | PDH, PFL, DRPA, POX, PPC | 0.53 | 0.39 | 0.0209 | b0114, b0902, b3951, b4381, b0871, b3956 | OBTFL | - |

Table S22: The maximum rates of heterologous lycopene growth-coupled production in iJO1366 (Strain2 in CM1 cultivation medium)

| **Number of knocked out reactions** | **Deleted reactions** | ***μ* (*h^-1^*)** | ***v_chemical_*** (mmol.gDW^-1^h^-1^) | **SSP (*h^-1^*)** | **Deleted genes** | **Co-Knockout reactions** | |
| --- | --- | --- | --- | --- | --- | --- | --- |
|  |  |  |  |  |  | **In the reduced model** | **Not in the reduced model** |
| **2** | PDH, DRPA | 0.53 | 0.39 | 0.0209 | b0114, b4381 | - | - |
| **3** | PDH, PGCD, PPM2 | 0.36 | 0.62 | 0.0228 | b0114, b2913, b4383 | - | - |
| **4** | PDH, DRPA, ACS, PGCD | 0.35 | 0.63 | 0.0224 | b0114, b4381, b4069, b2913 | ACCOAL | - |
| **5** | CBMKr, CBPS, PDH, DRPA, PSP_L | 0.33 | 0.64 | 0.0214 | b0521, b0323, b2874, b0032, b0114, b4381, b4388 | - | - |

Table S23: The maximum rates of the primary metabolites growth-coupled production in iJO1366 (Strain0 in CM2 cultivation medium)

| **Chemical** | **Number of knocked out reactions** | **Deleted reactions** | ***μ* (*h^-1^*)** | ***v_chemical_*** (mmol.gDW^-1^h^-1^) | **SSP (*h^-1^*)** | **Deleted genes** | **Co-Knockout reactions** | |
| --- | --- | --- | --- | --- | --- | --- | --- | --- |
|  |  |  |  |  |  |  | **In the reduced model** | **Not in the reduced model** |
| **succ** | **2** | F6PA, PYK | 0.17 | 6.45 | 0.1139 | b0825, b3946, b1854, b1676 | - | - |
|  | **3** | ACKr, PYK, F6PA | 0.10 | 9.19 | 0.0936 | b3115, b2296, b1849, b1854, b1676, b0825, b3946 | PPAKr, GART | - |
|  | **4** | ADPT, ALCD2x, LDH_D, PFL | 0.11 | 12.35 | 0.1391 | b0469, b1478, b1241, b0356, b2133, b1380, b0902, b3951 | ALCD19, OBTFL | LDH_D2 |
|  | **5** | ALCD2x, FADRx, PFL, LDH_D, THD2pp | 0.10 | 12.76 | 0.1333 | b1478, b1241, b0356, b3844, b0902, b3951, b2133, b1380, b1602 | ALCD19, FE3Ri, FLVRx, OBTFL | LDH_D2 |
| **akg** | **5** | THD2pp, PGL, ALDD2y, ME2, ABTA | 0.17 | 2.00 | 0.0345 | b1602, b0767, b3588, b2463, b2662, b1302 | - | ALDD3y |

Table S24: The guaranteed rates of succinate growth-coupled production in iJO1366 (Strain0 in CM1 cultivation medium)

| **Chemical** | **Number of knocked out reactions** | **Deleted reactions** | ***μ* (*h^-1^*)** | ***v_chemical_*** (mmol.gDW^-1^h^-1^) | | **Deleted genes** | **Co-Knockout reactions** | |
| --- | --- | --- | --- | --- | --- | --- | --- | --- |
|  |  |  |  | ***min*** | ***max*** |  | **In the reduced model** | **Not in the reduced model** |
| **succ** | **1** | FUM | 0.84 | 0.83 | 2.96 | b1612, b4122, b1611 | - | DTARTD |
|  | **2** | FUM, DHORD5 | 0.84 | 0.84 | 2.96 | b1612, b4122, b1611, 'b0945 | DHORD2 | DTARTD |
|  | **3** | CBMKr, CBPS, FUM | 0.79 | 1.26 | 3.47 | b0521, b0323, b2874, b0032, b1612, b4122, b1611 | - | DTARTD |
|  | **4** | ALATA_L, CBMKr, CBPS, FUM | 0.78 | 1.27 | 3.50 | b2290, b2379, b0521, b0323, b2874, b0032, b1612, b4122, b1611 | - | DTARTD |
|  | **5** | ACKr, ATPS4rpp, FUM, F6PA, PYK | 0.21 | 7.51 | 8.89 | b3115, b2296, b1849, b3736, b1612, b4122, b1611, b0825, b3946, b1854, b1676 | PPAKr, GART | DTARTD |

Table S25: The guaranteed rate of heterologous lycopene growth-coupled production in iJO1366 (Strain1 in CM1 cultivation medium)

| **Number of knocked out reactions** | **Deleted reaction** | ***μ* (*h^-1^*)** | ***v_chemical_*** (mmol.gDW^-1^h^-1^) | | **Deleted genes** | **Co-Knockout reactions** | |
| --- | --- | --- | --- | --- | --- | --- | --- |
|  |  |  | ***min*** | ***max*** |  | **In the reduced model** | **Not in the reduced model** |
| **5** | PDH, PFL, PPC, PPM, POX | 0.53 | 0.15 | 0.44 | b0114, b0902, b3951, b3956, b3380, b4383, b0871 | OBTFL, PPM2 | - |

Table S26: The guaranteed rates of heterologous lycopene growth-coupled production in iJO1366 (Strain2 in CM1 cultivation medium)

| **Number of knocked out reactions** | **Deleted reaction** | ***μ* (*h^-1^*)** | ***v_chemical_*** (mmol.gDW^-1^h^-1^) | | **Deleted genes** | **Co-Knockout reactions** | |
| --- | --- | --- | --- | --- | --- | --- | --- |
|  |  |  | ***min*** | ***max*** |  | **In the reduced model** | **Not in the reduced model** |
| **2** | PDH, PPM | 0.53 | 0.15 | 0.44 | b0114, b4383, b3380 | PPM2 | - |
| **3** | PDH, PGCD, PPM2 | 0.36 | 0.58 | 0.64 | b0114, b2913, b4383 | - | - |
| **4** | PDH, DRPA, ACS, PSP_L | 0.35 | 0.59 | 0.65 | b0114, b4381, b4069, b4388 | ACCOAL | - |
| **5** | CBMKr, CBPS, PDH, DRPA, PSP_L | 0.33 | 0.61 | 0.66 | b0521, b0323, b2874, b0032, b0114, b4381, b4388 | - | - |

Table S27: The guaranteed rates of succinate growth-coupled production in iJO1366 (Strain0 in CM2 cultivation medium)

| **Chemical** | **Number of knocked out reactions** | **Deleted reaction** | ***μ* (*h^-1^*)** | ***v_chemical_*** (mmol.gDW^-1^h^-1^) | | **Deleted genes** | **Co-Knockout reactions** | |
| --- | --- | --- | --- | --- | --- | --- | --- | --- |
|  |  |  |  | ***min*** | ***max*** |  | **In the reduced model** | **Not in the reduced model** |
| **succ** | **3** | ALCD2x, LDH_D, PFL | 0.11 | 7.28 | 12.68 | b1478, b1241, b0356, b2133, b1380, b0902, b3951 | ALCD19, OBTFL | LDH_D2 |
|  | **4** | ALCD2x, GLUDy, PFL, LDH_D | 0.10 | 7.36 | 12.76 | b1478, b1241, b0356, b1761, b0902, b3951, b2133, b1380 | ALCD19, OBTFL | LDH_D2 |
|  | **5** | ALCD2x, FADRx, PFL, LDH_D, THD2pp | 0.10 | 10.29 | 12.97 | b1478, b1241, b0356, b3844, b0902, b3951, b2133, 'b1380, b1602 | ALCD19, FE3Ri, FLVRx, OBTFL | LDH_D2 |
